# Recent advances in aptamer-based biosensing technology for isolation and detection of extracellular vesicles

**DOI:** 10.3389/fcell.2025.1555687

**Published:** 2025-07-23

**Authors:** Osama Alnaser-Almusa, Mohammed Mahmoud, Mohammed Ilyas, Raghda Adwan, Farah Abul Rub, Noha Alnaser-Almusa, Fayrouz Mustafa, Sana Ahmed, Alaa Alzhrani, Tanveer Ahmad Mir, Mubarak. Alabudahash, Raja Chinnappan, Ahmed Yaqinuddin

**Affiliations:** ^1^ College of Medicine, Alfaisal University, Riyadh, Saudi Arabia; ^2^ Laboratory of Tissue/Organ Bioengineering & BioMEMS, Organ Transplant Centre of Excellence (TR&I-Dpt), King Faisal Specialist Hospital & Research Centre, Riyadh, Saudi Arabia; ^3^ Department of Medical Laboratory Technology, Faculty of Applied Medical Sciences, King Abdulaziz University, Jeddah, Saudi Arabia; ^4^ Pharmaceutical Analysis Department, Strathclyde Institute of Pharmacy and Biomedical Sciences (SIPBS), Glasgow, United Kingdom

**Keywords:** aptasensors, aptamers, extracellular vesicles, exosomes, SELEX, EVs isolation and detection

## Abstract

Since their discovery in the 1970s, extracellular vesicles (EVs) have garnered significant scientific attention due to their involvement in diverse pathological processes, including tumorigenesis. Their unique properties have also piqued interest for various applications such as transporting biomolecules for drug delivery. Researchers have developed numerous isolation and detection methods for EVs, including ultracentrifugation, immunoaffinity capture, and antibody-based biosensors. However, these techniques often suffer from limitations in sensitivity, specificity, and efficiency, hindering their performance and reliability in research and clinical settings. Aptamers are short, single-stranded DNA or RNA molecules created to selectively bind to a specific target and offer a promising alternative to antibodies. These aptamers are identified by a process known as SELEX. By repeatedly selecting and amplifying aptamers with high binding affinity, SELEX can generate aptamers with exceptional specificity and sensitivity. Aptamers can then be incorporated into biosensors, known as aptasensors, for EV isolation, detection, and analysis. Aptasensors offer several advantages over antibody-based methods. They are often more stable, can be produced synthetically at lower cost, and can be easily modified for various applications. Additionally, aptamers can be selected against a wide range of targets, including proteins, nucleic acids, and small molecules, making them versatile tools for EV research. This review discusses various SELEX methods for aptamer detection, the clinical uses of aptamers, and the types of EV analysis methods.

## 1 Introduction

### 1.1 Extracellular vesicles (EVs)

The field of extracellular vesicles (EVs) has developed expanding over the past few decades as researchers have increasingly recognized their biological significance. In the early 2000s, scientists believed that EVs played limited roles, mainly in intercellular communication, tumor progression, and tissue repair. However, it is now evident that EVs play crucial roles in various physiological and pathological processes, serving as diagnostic biomarkers and therapeutic agents—particularly in the form of exosomes and microvesicles (Yáñez-Mó et al., 2015). Extracellular vesicles (EVs) are cell-derived membrane carriers and there is interest in EVs due to their potential importance in intercellular communication through the exchange of RNA, DNA, lipids, and proteins (Kalluri and LeBleu, 2020; Nowak et al., 2023). The importance of EVs lies in their ability to convey information to other cells, thereby affecting how the recipient cell functions (Foster et al., 2016; Elsharkasy et al., 2020). The distinctive packaging of this information offers both protection and the ability to deliver multiple messages simultaneously, even to distant sites from where the vesicle originated. Furthermore, they can selectively adhere to specific cells or tissues through receptor-mediated interactions, facilitating the targeted release of their contents into corresponding structures (Yáñez-Mó et al., 2015). Hence, they play a crucial role in intercellular communication (Harding et al., 1983; Pan et al., 1985). EVs are released from the surfaces of normal, cancerous, and apoptotic cells and are present in various body fluids such as saliva, urine, milk, and amniotic fluid (Chang et al., 2021). The current classification of EVs primarily categorizes them based on size and their biogenesis. EVs can be broadly classified into three main types: exosomes, microvesicles, and apoptotic bodies (Sedgwick and D’Souza-Schorey, 2018; Szwedowicz et al., 2022).

The biogenesis of microvesicles (MVs), a subtype of EVs, is a complex and multifaceted process that involves the interplay of various cellular components and signaling pathways. Microvesicles, originally identified from activated blood platelets and erythrocytes for their role in coagulation bud directly from the plasma membrane at specific sites by alterations in protein and lipid composition and elevation in Ca^2+^ levels, followed by fission, and release into the extracellular space (Aatonen et al., 2014; Minciacchi et al., 2015). Elevated Ca^2+^ levels in the plasma membrane lead to activation of calcium-dependent enzymes like scramblase and floppase that modify membrane lipid composition (Piccin et al., 2007). MVs are enriched with lipid raft domains; therefore, their formation can be hindered by cholesterol depletion (Del Conde et al., 2005). Proteins involved in maintaining cell shape through actin dynamics regulation also contribute to MV biogenesis (Crespin et al., 2009; Li et al., 2012). RhoA, which is a small GTPase protein involved in cytoskeleton regulation, along with its downstream effectors ROCK and LIMK, regulates MV release, and well as Calpain, which is a calcium-dependent enzyme in platelets, that plays a role in MV biogenesis. ARF6 identified as crucial in MV formation, regulates an endosomal complex that selectively incorporates cargo into MVs. Downstream targets of ARF6, ERK, and MLCK influence actin dynamics and myosin activity, which are critical for MV release. Inhibiting ARF6 or its targets reduces MV release into the extracellular space (D’Souza-Schorey and Chavrier, 2006; Muralidharan-Chari et al., 2009).

**FIGURE 1:**
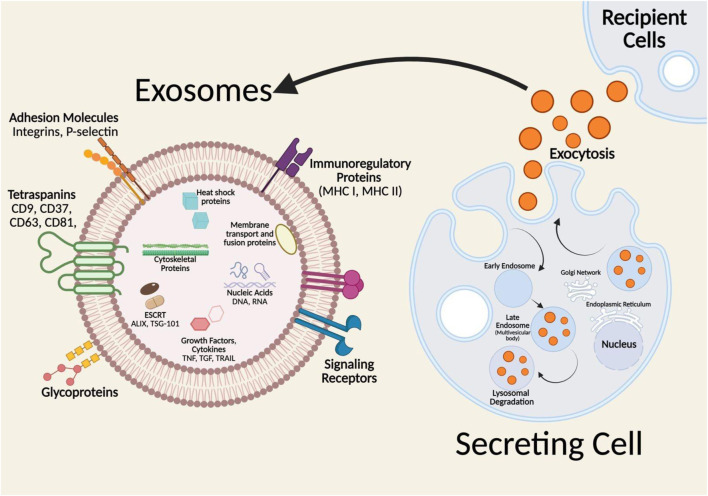
Formation and molecular composition of exosomes. Insert: represents the lipid bilayer structure of exosomes and associated biomarkers transported to the recipient cells. Adopted from ref Tenchov et al. (2022), with copyright permission under the terms of the CC-BY-NC-ND 4.0 license. Produced by Biorenders.

On the other hand, the exosome biogenesis process begins with the transport of molecular cargo into the cell. The early endosome, generated by the plasma membrane budding inward, is the first stage in the endosomal trafficking route, sorting and determining the fate of the endocytosed cargo (Figure 1) (Woodman and Futter, 2008; Grant and Donaldson, 2009; Krylova and Feng, 2023). The cargo can exit the early endosome by one of three pathways: recycling and endosomal maturation, lysosomal destruction, or exosome release. Cargo that needs to be recycled will localize to the endosomes peripheral tubular domains and then dissociate to fuse with the Golgi network or the plasma membrane in the recycling endosome. Cargo not intended for recycling will cluster at the central vacuolar areas of the early endosome and commit to the endosomal maturation pathway, eventually forming late endosomes. Late endosomes will either merge with lysosomes for destruction or produce intraluminal vesicles with the plasma membrane (ILVs) known as exosomes (Woodman and Futter, 2008). During the endosomal maturation process, the endosomal membrane composition changes, with sphingomyelin replaced by ceramides and the early endosome marker Rab5 replacing the late endosome marker Rab11 (Sönnichsen et al., 2000; Megha and London, 2004). As the endosome grows, specific sections of its membrane begin to invade and branch away from the cytoplasm into the intraluminal space, generating ILVs. The late endosomes containing these intraluminal vesicles (ILVs) are known as multivesicular bodies (MVBs). If an MVB fuses with the lysosome, the cargo within the ILVs is destroyed, however, if an MVB fuses with the cell’s plasma membrane, the ILVs are released into the extracellular environment and become exosomes (van Niel et al., 2011; Aatonen et al., 2014).

Apoptotic bodies are formed during the apoptosis process which is a major mechanism of cell death for both normal and cancerous cells (Kerr et al., 1972; Akers et al., 2013). Apoptosis causes a cell to die in many phases, beginning with nuclear chromatin condensation, followed by membrane blebbing possibly mediated by actin-myosin interaction, and finally breakdown of the cellular content into separate membrane-enclosed vesicles known as apoptotic bodies or apoptosomes (Coleman et al., 2001; Elmore, 2007). The clearance of apoptotic bodies by macrophages via phagocytosis is governed by particular interactions between phagocyte recognition receptors and alterations in the apoptotic cell membrane. These alterations include the oxidation of surface molecules, which creates sites for Thrombospondin (Tsp), which is a glycoprotein that associates with the extracellular matrix and has roles in cell signaling and tissue remodeling, and the complement protein C3b binding (Sebbagh et al., 2001).

Cancer-derived EVs have been shown to significantly impact the tumor microenvironment and drive cancer progression. These EVs contain a variety of tumor-specific molecules, including proteins and RNAs, which further contribute to the malignant nature of cancer. Understanding the precise mechanisms by which cancer EVs mediate intercellular communication in patients could lead to the development of novel cancer treatment strategies. Additionally, analyzing the contents of cancer EVs found in body fluids may enable their use as clinically valuable diagnostic and prognostic biomarkers, as well as potential therapeutic targets (Urabe et al., 2020).

### 1.2 Traditional EV detection methods

Currently, several methods are employed for the isolation of exosomes, primarily based on size, density, and surface protein expression. Common techniques include differential centrifugation, density gradient centrifugation, size exclusion chromatography, and polymer-based precipitation and immunoaffinity capture like ELISA. Ultracentrifugation is a widely used technique for isolating EVs which leverages high centrifugal forces to separate EVs based on their size and density (Momen-Heravi, 2017). Cells, cell debris, apoptotic bodies, and biopolymer aggregates are the first particles to settle. To reduce losses from co-sedimentation and contamination with cell lysis products, this step includes several substeps, including centrifugation at 300–400 × g for 10 min to sediment a main portion of the cells, at 2000 × g to remove cell debris, and at 10,000 × g to remove aggregates of biopolymers, apoptotic bodies, and other structures with buoyant density higher than that of EVs. EVs in the supernatant are sedimented by ultracentrifugation at >100,000 × g (100,000–200,000 × g) for 2 h. The non-EV proteins in the EV pellet are suspended and then ultracentrifuged repeatedly (Théry et al., 2006). The EV preparation is purified and separated microparticles are selected based on their size using microfiltration of suspension employing filters with pore sizes of 0.1, 0.22, or 0.45 μm (Van Deun et al., 2014; Zarovni et al., 2015; Xu et al., 2016). Size exclusion chromatography (SEC) is an effective technique for isolating EVs from complex biological samples. This method separates particles based on their size, allowing for the efficient removal of smaller contaminants, such as proteins and lipoproteins, while preserving the integrity of the EVs. SEC operates on the principle that larger particles elute from the column before smaller ones. This allows for the separation of EVs from smaller soluble proteins and other contaminants. Moreover, SEC columns are typically filled with porous beads made from materials like agarose or dextran. The pore size of these beads is crucial, as it determines the size exclusion limit and influences the separation efficiency (Huang and He, 2017).

Immunoaffinity capture is a specialized technique used for isolating EVs by exploiting the specific interactions between antibodies and common surface proteins on the EVs such as CD9, CD63, and CD81. This method allows for the targeted enrichment of particular EV subpopulations based on their unique surface markers, enhancing the purity and homogeneity of the isolated vesicles (Huang et al., 2021; Fortunato et al., 2022). ELISA-Based Immunoaffinity Capture is a powerful technique for isolating Evs by utilizing specific antibodies that are coated onto the wells of an ELISA plate, allowing for the selective capture of EVs that express particular surface markers. Once the EVs are captured, they can be subjected to both quantitative and qualitative analyses, to assess the presence and abundance of EV-associated proteins (Brambilla, 2022).

### 1.3 Limitations of traditional methods

Traditional methods for detecting EVs present several significant limitations that impact their reliability and effectiveness in research and clinical applications. One major challenge is the sensitivity of conventional detection techniques, which often struggle to identify low-abundance EVs amidst a complex biological background, leading to potential underrepresentation of these vesicles in analyses (Shami-Shah et al., 2023). Additionally, many traditional methods, such as Western blotting and ELISA, require substantial quantities of EVs for adequate sensitivity, which can be problematic given that EVs are often present in limited amounts in biological samples (Davidson et al., 2023). Another limitation is the inability to accurately characterize EVs due to their heterogeneity in size, composition, and origin. Traditional isolation techniques may not effectively distinguish EVs from other particles, such as lipoproteins and protein aggregates, resulting in contamination that complicates downstream analyses. Furthermore, the lack of standardized protocols across different laboratories leads to variability in results, making it difficult to compare findings or draw definitive conclusions (Zhao Z. et al., 2021).

Quantification challenges also arise, as many traditional methods do not provide sufficient information to accurately assess EV concentration and purity. This is exacerbated by the absence of reference proteins in EV samples, which complicates normalization in immunoblotting experiments (Davidson et al., 2023) Moreover, traditional techniques often involve multiple steps that can introduce variability and increase the risk of sample loss or degradation, particularly of sensitive biomolecules like RNA. Finally, the technical complexity and time-consuming nature of these methods can hinder their scalability and throughput, limiting their application in high-throughput settings or clinical diagnostics (Gandham et al., 2020). Overall, while traditional EV detection methods have contributed to our understanding of EV biology, their limitations underscore the need for more advanced, sensitive, and standardized techniques to fully exploit the potential of EVs in biomedical research and clinical applications. Therefore, there is a pressing need for fast, reliable, and scalable platforms for the detection and isolation of EVs for diagnostic and therapeutic applications. Aptamer-based biosensing is a promising technique that addresses these requirements by offering high specificity and affinity for target molecules. Aptamers possess several advantageous properties, including high stability, scalability, and the ability to be easily integrated into biosensing platforms. The application of aptamer-based biosensing presents a transformative approach toward clinically viable EV-based diagnostics and therapeutics (Sequeira-Antunes and Ferreira, 2023).

## 2 Biosensors

**FIGURE 2:**
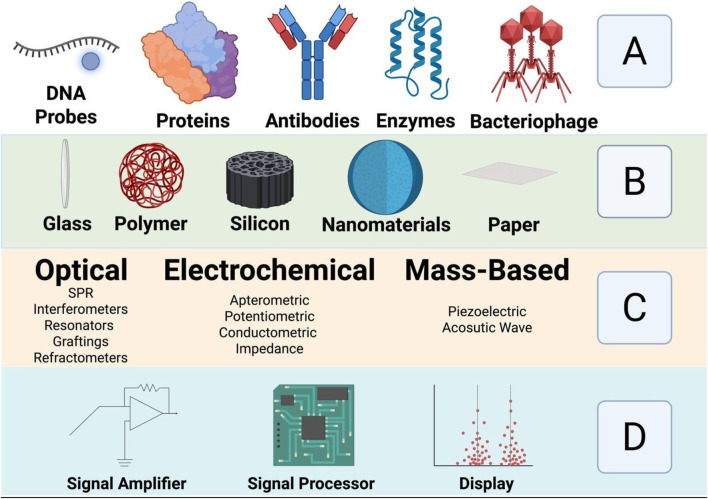
Shows schematically the different parts of a biosensor: **(A)** biorecognition molecules **(B)** Sensing platform **(C)** Transducer types and **(D)** Electronic part. Adopted from ref Roointan et al. (2019), with copyright permission. Produced by Biorenders.

Biosensors are analytical devices that combine a biological recognition element with a physicochemical transducer to detect and quantify specific analytes (Cammann, 1977; Newman and Turner, 2005). Figure 2 is a schematic showing the different parts of a biosensor. The recognition element, which can be an enzyme, antibody, nucleic acid, aptamer, or other biologically active material, specifically interacts with the target analyte. The transducer then converts this interaction into a quantifiable signal, often electrical, optical, or thermal, which can be further processed and displayed (Rodriguez-Mozaz et al., 2005).

**FIGURE 3:**
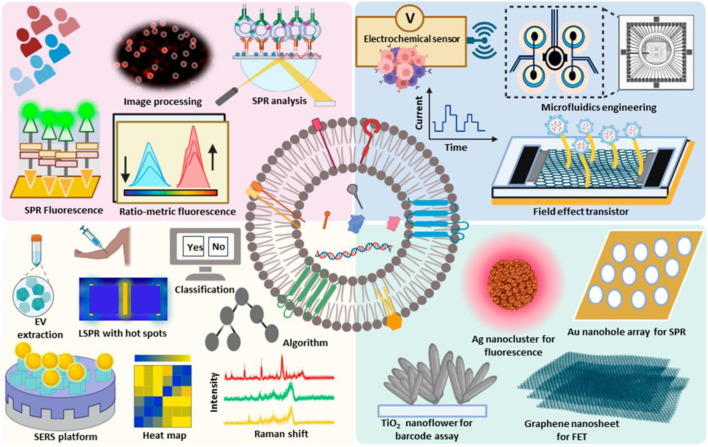
Extracellular vesicles possess cellular components such as lipids, and genetic materials such as RNA and nucleic acids and act as valuable markers for cancer, and other diseases. The advanced technologies developed for the detection and diagnosis of extracellular vesicles have been depicted. Reproduced from ref Das et al. (2024), with copyright permission under the terms of the CC-BY-NC-ND 4.0 license.

Biosensors have revolutionized the field of extracellular vesicle (EV) detection due to their intrinsic advantages. One of their primary benefits is high sensitivity, which allows for the detection of low concentrations of EVs. EVs, which include exosomes and microvesicles, play a significant role in intercellular communication and are considered potential biomarkers for various diseases, including cancer and neurodegenerative disorders (Reiner et al., 2017; Das et al., 2024) (Figure 3). The implication of EVs as biomarkers for different diseases makes their detection crucial for early diagnosis and monitoring of diseases (Simons and Raposo, 2009). Another key advantage of biosensors is their capability for detection. Traditional methods for detecting EVs require arduous labeling steps, such as fluorescence or radioactivity, which can alter the properties of the EVs and introduce artifacts into the analysis. In contrast, label-free biosensors detect the analyte directly. This allows the preservation of the native state of EVs and enables more accurate analysis (Fan et al., 2008; Peltomaa et al., 2018). An example of a technique which uses detection is surface plasmon resonance (SPR) and quartz crystal microbalance (QCM), which have been successfully used in EV detection (Min et al., 2020).

Portability is another advantage of biosensors. The miniaturization of biosensor components has led to the development of portable devices that can be used for point-of-care testing, making them accessible in various settings, including clinics and fieldwork. This portability is particularly important in resource-limited environments where access to advanced laboratory facilities may be restricted. Portable biosensors also allow for real-time monitoring of EVs, providing immediate results that are critical in clinical decision-making (Srinivasan and Tung, 2015; Soleymani and Li, 2017). Additionally, biosensors can be integrated with microfluidic systems, which enable the manipulation of small fluid volumes and high-throughput analysis (Luka et al., 2015). This integration is particularly beneficial for the detection of EVs in complex biological samples, such as blood, urine, or saliva, where the concentration of EVs may be low and sample availability is limited. Microfluidic-based biosensors, referred to as lab-on-a-chip devices, combine multiple analytical processes on a single chip, which enhances the efficiency and accuracy of EV detection (Guo et al., 2018).

Recent advances in nanotechnology have further improved the performance of biosensors. The incorporation of nanomaterials, such as gold nanoparticles, carbon nanotubes, and quantum dots, into biosensor designs has significantly enhanced their sensitivity and specificity (Kim et al., 2022). For example, gold nanoparticles can amplify the signal generated by the bioreceptor-analyte interaction, enabling the detection of EVs at extremely low concentrations (Reiner et al., 2017).

### 2.1 Antibody-based biosensors for EVs

The recognition element used in a biosensor determines the specificity and selectivity of the biosensor, which enables it to respond to its specific target. Therefore, the recognition element is chosen based on the target of interest; for example, antibodies and aptamers are appropriate for detecting pathogens, while enzymes are more fit for catalytic reactions (Kissinger, 2005; Datta et al., 2013). Antibodies have recently become a widely used recognition element in biosensors because the target of interest (the immunogen) does not require purification before detection (Saerens et al., 2008). Recombinant antibodies have also been created by genetic modification of antigen-binding sites of common antibodies (Emanuel et al., 2000). In the context of EV detection, antibodies can be employed to selectively bind to surface proteins or other molecular markers present in EVs. This binding event is then transduced into a measurable signal, which can then be used for quantification and analysis of EVs in biological samples (Gaillard et al., 2020).

One of the most common methods utilizing antibodies for EV detection is the electrochemical immunosensor. In this approach, antibodies are immobilized on an electrode surface, where they capture EVs from the sample. The binding of EVs to the antibodies induces an electrochemical signal that is directly proportional to the concentration of EVs in the sample (Doldán et al., 2016). Electrochemical immunosensors are known for their high sensitivity and specificity, making them suitable for detecting low-abundance EVs in complex biological fluids (Grieshaber et al., 2008). Another widely used method is surface plasmon resonance (SPR) biosensing. In SPR-based sensors, antibodies are immobilized on a gold surface, and the binding of EVs to these antibodies induces changes in the refractive index near the sensor surface, resulting in a detectable SPR signal (Reiner et al., 2017). SPR biosensors are highly effective for studying the dynamics of EV interactions because they offer real-time monitoring and detection (Min et al., 2020).

Flow cytometry is another technique commonly used for EV detection that involves the use of fluorescently labeled antibodies. This method utilizes fluorescently labeled antibodies that specifically bind to surface markers on EVs, allowing for the simultaneous analysis of multiple markers. The ability to label and detect multiple antigens on individual EVs provides a comprehensive view of the heterogeneity and composition of EV populations, which is essential for understanding their biological roles and potential as biomarkers in various diseases. Flow cytometry’s high-throughput capability is another significant advantage, enabling the analysis of large numbers of EVs in a relatively short amount of time. This makes it a valuable tool for both research and clinical applications where understanding the diversity of EVs is crucial, such as in cancer diagnosis or monitoring the progression of diseases (Görgens et al., 2019). However, detecting vesicles using side scatters is challenging due to their small size, and it is further complicated by the ‘swarm effect,’ where multiple vesicles are mistakenly identified as a single event. This makes it difficult to determine whether multiple markers are expressed on the same individual vesicle.

Western blotting is also frequently employed in EV research for the detection of specific proteins on EV membranes. In this technique, EVs are first separated by gel electrophoresis, followed by the transfer of the proteins to a membrane where they are probed with specific antibodies. The presence of the target protein is then visualized using chemiluminescence or other detection methods (Kowal et al., 2017). Western blotting is widely used in EV research for detecting specific proteins on EV membranes, particularly those that serve as markers like CD63, CD81, and CD9. In this technique, EV proteins are separated by SDS-PAGE, transferred to a nitrocellulose or PVDF membrane, and then probed with specific antibodies (Suck and Krupinska, 1996; Penna and Cahalan, 2007; Jiang et al., 2019).

Biosensors can be integrated with microfluidic systems. An example of this technology utilizes antibodies as the recognition element, and this has been demonstrated by using the Surface Plasmon Resonance Imaging (SPRi) method to detect breast cancer (Lee et al., 2007). This integration allows for the real-time, detection of breast cancer biomarkers, such as HER2, with high sensitivity and specificity. The microfluidic system enables precise control of sample flow and enhances the interaction between the antibodies and the target biomarkers, leading to improved detection limits.

### 2.2 Disadvantages of antibody-based biosensors

Despite their widespread use, antibody-based biosensors have several limitations that can impact their performance and reliability. One of the primary limitations is the high cost associated with the production and purification of antibodies. This cost can be prohibitive, especially in large-scale studies or clinical applications where significant quantities of antibodies are required (Klutz et al., 2016). Another significant limitation of antibody-based biosensors is the batch-to-batch variability that can occur during antibody production. This variability can lead to inconsistencies in the performance of biosensors, particularly affecting their sensitivity and specificity. Such variability is a critical issue in clinical diagnostics, where reproducibility and precision are essential for reliable results (Ferrigno, 2016). Additionally, antibodies are proteins that can degrade over time, leading to reduced stability and a shorter shelf life of the biosensors. This instability necessitates careful storage and handling, which can add complexity to their use (Luan et al., 2018).

**FIGURE 4:**
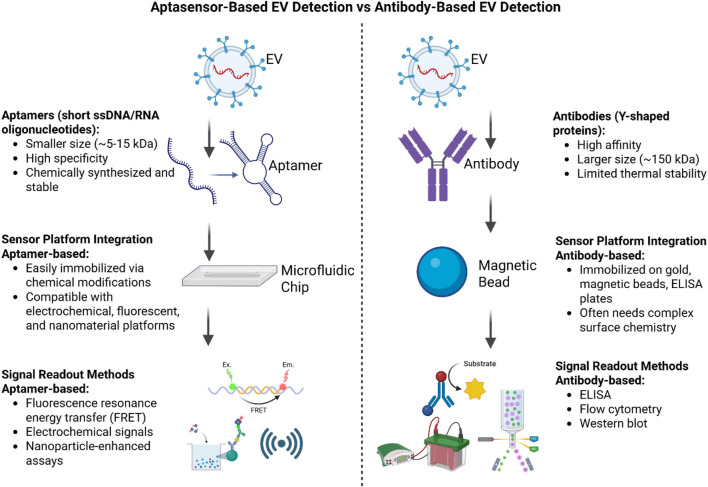
Comparison of antibody-based and aptamer-based (aptasensor) methods for extracellular vesicle (EV) detection. The illustration highlights differences in recognition elements, sensor platform integration, signal readout methods, advantages, and limitations, where ptamer-based methods are more stable, easier to synthesize, and more adaptable, while antibody-based methods are established but less stable and harder to produce.

While antibodies are valuable tools for biosensors, they have several limitations that highlight the need for alternative recognition elements, such as aptamers, which offer greater stability and higher affinities (Crivianu-Gaita and Thompson, 2016) and also highlighted in Table 1. As illustrated in Figure 4, aptamer-based EV detection offers advantages over antibody-based methods, including higher chemical stability, lower production costs, easier modification, and better batch-to-batch consistency, making it more robust, scalable, and accessible for microfluidics and electrochemical biosensing.

**TABLE 1:** Advantages of aptamers over monoclonal antibodies (Aljohani et al., 2022).

Monoclonal antibody	Aptamers
Proven Immunogenicity	Non-Immunogenic
Expensive Synthesis Process	Cost-effective Synthesis
Inefficient cellular uptake	Rapid entry into cells
Bacterial or Viral Intrusion in production can degrade product quality	The chemical manufacturing process is free from biological contamination risks
Clonal heterogeneity	No clonal heterogeneity
Restricted potential for chemical modification	Extensive potential for chemical modification
Restricted capacity to apply negative selection pressure	Capability for negative selection
Hard to modify	Readily adjustable

## 3 Aptamers

Aptamers are a group of synthetic oligomers or short single-stranded nucleic acids, DNA or RNA, typically consisting of 20–100 nucleotides (Ohuchi, 2012; Guan and Zhang, 2020). They are designed to bind to specific target molecules or ligands by folding into a three-dimensional conformation in aqueous solutions (Nimjee et al., 2017). This folding is achieved through various bonds such as electrostatic interactions, van der Waals forces, and hydrogen bonding (Rozenblum et al., 2016). These bonds or loops create motifs with high affinity and binding capability to targeted ligands (Adachi and Nakamura, 2019). Aptamers can be designed in many different configurations based on their intended use, such as loops, pseudoknots, bulges, hairpins, quadruplexes, and double helix structures. Additionally, these aptamers can be used to inhibit or activate their targets (Patel et al., 1997).

The premise of creating aptamers was to find a replacement for antibodies because they cause immunogenicity (Yan and Levy, 2009). Additionally, since aptamers are synthetic molecules, they are more efficient, less costly, and have more utilities than other options. These molecules are known as “chemical antibodies” and “nucleic acid versions of antibodies”. Aptamers have lower molecular weight, are nontoxic, and have a higher affinity for binding to target molecules (Ng et al., 2012; Chinnappan et al., 2020b). The major advantage of aptamers is that they are reusable as they can be regenerated after binding to their targets (Ku et al., 2015). They are very thermostable, so they are resilient to harsh environments such as extreme temperatures, pH, and humidity (Liu et al., 2022). They are easily amplified using PCR and do not need post-translational chemical modifications to function appropriately. Thus, the use of aptamers is expanding beyond a laboratory diagnostic tool; they are currently being used as biomarkers, bioimaging agents, drug deliverers, and in many other useful diagnostic and therapeutic functions as described in Figure 4 (Chinnappan et al., 2020a; Chinnappan et al., 2023a). However, a major issue with using aptamers is that the success rate of procedures using them is lower. This is mainly due to the structural complexity of these manufactured molecules compared to antibodies (Kohlberger and Gadermaier, 2022). Fortunately, there have been efforts to address these issues using specialized SELEX techniques and integrating stricter quality control processes while creating these molecules, so now once an aptamer is identified, it can be easily regenerated, resulting in consistent performance when using them (Famulok and Mayer, 2014).

**FIGURE 5:**
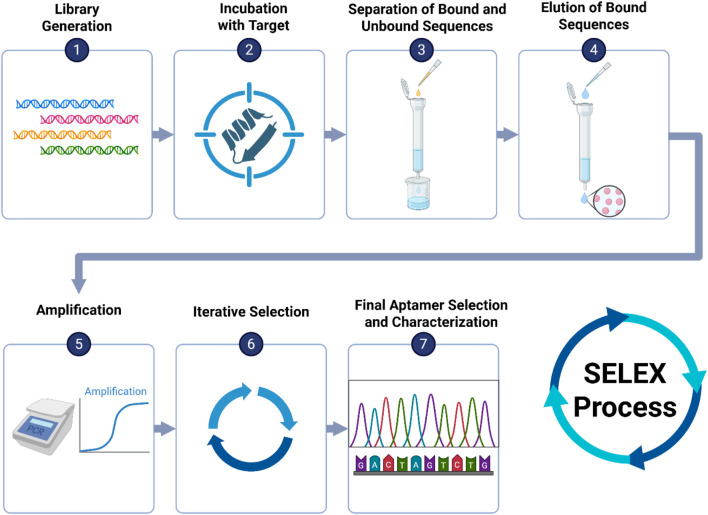
Overview of the SELEX process for aptamer selection: (1) Generate a large oligonucleotide library with randomized regions. (2) Incubate with the target (e.g., protein, virus). (3) Separate bound from unbound sequences using membrane filtration or affinity chromatography. (4) Elute bound sequences. (5) Amplify selected sequences via PCR or RT-PCR. (6) Repeat selection cycles to enrich high-affinity binders. (7) Sequence and characterize final aptamer candidates.

Since aptamers form stable three-dimensional structures, they can be designed using computer algorithms for sequence-based modeling. This *in vitro* production eliminates the need for animal involvement, enabling the creation of aptamers against proteins that are endogenous to antibodies, which can be challenging to target with traditional methods (Radom et al., 2013). Aptamers are generated through Systematic Evolution of Ligands by Exponential Enrichment (SELEX) (Eissa et al., 2020). SELEX is a process for selecting aptamers against any chosen target, such as proteins, bacteria, viruses, or cells (Tan et al., 2016; Chinnappan et al., 2017; Chinnappan et al., 2020c). The process involves repetitive cycles of incubating the DNA or RNA pool with the target molecule, binding the DNA or RNA strands to the target, separating the binders from the target molecules using membrane filtration or affinity chromatography, and selectively amplifying high-affinity binders using PCR or RT-PCR (Kaur, 2018) as shown in Figure 5.

Even though DNA and RNA aptamers have great applications in diagnostics and therapeutics, their utility is still limited. This mainly stems from their fixed ribose and deoxyribose structures. Therefore, newer approaches to selecting more efficient aptamers from xenobiotic nucleic acid (XNA) libraries are emerging (Chaput and Herdewijn, 2019). Synthetic XNA backbones are fundamentally very different from natural nucleic acids, so they are created in a way to become more resistant to hydrolyzing or deprotonation effects of nucleases in the body (Lozoya-Colinas et al., 2023). The problem with XNA aptamers, however, is that they require specialized SELEX techniques known as X-SELEX for their production. X-SELEX has steps similar to conventional SELEX such as incubation and amplification, but it also involves the transcription of DNA libraries into XNA polymers by DNA-dependent XNA polymerases (Taylor and Holliger, 2018). This process is more demanding than conventional SELEX; however, it yields a new variety of aptamers that could perform unique functions such as the 2′-deoxy-2′-fluorarabino nucleic acid-based aptamer that has a high affinity for HIV-1 reverse transcriptase or the 2′-O-methyl-ribose–1,5 anhydrohexitol nucleic acid (MeORNA–HNA) aptamers against rat VEGF (Alves Ferreira-Bravo et al., 2015; Eremeeva et al., 2019).

While advances like XNA-based aptamers continue to enhance the chemical variety and robustness of these molecules, it is also vital to highlight how aptamers are already being used in real-world therapeutic contexts (Lozoya-Colinas et al., 2023). In the context of HIV, specifically regarding Pre-Exposure Prophylaxis (PrEP), a Tenofovir (TFV) aptasensor was utilized to assess drug adherence through the detection of TFV levels in bodily fluids. The trial (NCT04870671) was completed, successfully detecting the drug in saline samples; however, it did not yield reliable results in plasma due to protein interference. Aptamers were employed in the context of bladder cancer, leading to the development of electro-phage and aptamer sensors for the detection of urinary biomarkers in cancer patients. The ongoing trial (NCT02957370) has advanced to the development of aptamer and phage probes, with clinical validation in progress. These examples demonstrate the varied applications of aptamers in diagnostics and monitoring within various clinical contexts.

### 3.1 Aptamer selection techniques

Significant efforts have been dedicated to enhancing selection efficiency in SELEX by tailoring methods to specific targets and screening techniques (Lin et al., 2024). This has been instrumental in developing high-performance aptamers that address the limitations of traditional SELEX. Advancements in various SELEX domains have spurred the creation of innovative separation and enrichment techniques to isolate functional aptamers. Additionally, optimizing selection conditions and leveraging high-throughput sequencing with bioinformatics have enabled researchers to predict evolutionary trends, reduce screening rounds, and mitigate sequence loss during repetitive processes (Song et al., 2013; Li L. et al., 2021). SELEX is a gold standard technology for generating nucleic acid aptamers. Recent advancements have expanded its application beyond nucleic acids to include targets such as recombinant proteins, cell surface proteins, extracellular vesicles, and even whole cells (Darmostuk et al., 2015; Sun et al., 2023). The repertoire of SELEX methods has grown to encompass cell SELEX, capillary electrophoresis SELEX, magnetic bead-based SELEX, microfluid SELEX, and capture SELEX, among others as discussed in Table 2 (Yang and Bowser, 2013; Hung et al., 2014; Duan et al., 2022).

**TABLE 2:** SELEX methods.

SELEX method	Key feature	Advantages	Limitations	Reference
Conventional SELEX	Incubates target protein (e.g., IgE) with ssDNA in tubes, then separates with beads and amplifies with PCR.	Simple setup, well-established, widely used; compatible with many targets; strong binding interactions possible	Random protein immobilization; decreased selection stringency; several PCR and ssDNA conversion processes	Chinnappan et al. (2021), Meng et al. (2023)
Negative SELEX	Uses immobilization matrix alone to remove non-specifically binding sequences	Improves specificity by eliminating matrix binders	Requires extra selection step	Zhuo et al. (2017)
Counter SELEX	Incubation with structurally similar targets to remove cross-reactive sequences	Enhances specificity against structurally similar non-targets	Needs known similar non-target molecules	Jenison et al. (1994), Zhuo et al. (2017)
CE-SELEX	Capillary electrophoresis separates bound and unbound sequences based on mobility differences	Highly efficient, reduces rounds to 1–4; high-affinity aptamers	Limited injection volume; requires CE equipment	Mendonsa and Bowser (2004)
Non-SELEX (NECEEM)	Avoids PCR; uses non-equilibrium capillary electrophoresis to separate complexes	Fast (∼1 h); avoids PCR bias	Smaller library size; limited by capillary capacity	Ashley et al. (2012)
μFFE-SELEX	Uses micro free-flow electrophoresis to increase library capacity	Larger library size (∼10^14^); better coverage of sequence space	Specialized equipment required; low success rate	Jing and Bowser (2011)
Microfluidic SELEX	Integrates microfluidics with SELEX (magnetic beads, sol-gels, chips)	Automated, reduced reagent use, selection in fewer rounds	Bead aggregation, flow disruption, device complexity	Hybarger et al. (2006), Dembowski and Bowser (2018)
Cell SELEX	Whole live cells used as selection target	Targets proteins in native state; no prior knowledge needed	Higher complexity, cell variability	Daniels et al. (2003), Graham and Zarbl (2012)
Hybrid/TECS SELEX	Combines cell and purified protein SELEX, or uses surface-displayed proteins	Allows targeting of hard-to-purify proteins	Needs recombinant expression systems	Soldevilla et al. (2016), Zhuo et al. (2017)
*In Vivo* SELEX	Performed in living organisms for functional aptamer discovery	Physiologically relevant aptamers; crosses barriers like BBB	Complex, resource-intensive	Mi et al. (2010), Sola et al. (2020)
HTS-SELEX	Applies high-throughput sequencing across SELEX rounds	Detects enrichment early, fewer rounds needed, rich data for bioinformatics analysis	Requires HTS access and computational analysis	Nguyen Quang et al. (2016), Pantier et al. (2022)

Abbreviations: SELEX, Systematic Evolution of Ligands by EXponential enrichment; CE., capillary electrophoresis; NECEEM, Non-Equilibrium Capillary Electrophoresis of Equilibrium Mixtures; μFFE, Micro Free-Flow Electrophoresis; HTS, High-Throughput Sequencing; TECS, Target-Expressing Cell SELEX; BBB, Blood-Brain Barrier.

Among these methods, Capillary Electrophoresis SELEX (CE-SELEX) strikes an ideal balance between efficiency, specificity, and fewer selection cycles. Unlike regular SELEX, which can take up to 15–20 rounds, CE-SELEX can isolate high-affinity aptamers in as little as 1-4 rounds, significantly reducing development time and expenses (Brown et al., 2024). Furthermore, CE-SELEX does not need immobilization of the target, keeping its native structure—an important consideration when working with fragile or complicated proteins. Its capacity to discriminate bound and unbound sequences via electrophoretic mobility improves the quality of aptamer candidates. However, the method’s disadvantages, such as decreased sample throughput and the requirement for specialized equipment, may render it inappropriate for all applications. Nonetheless, CE-SELEX is still one of the most efficient and clean procedures for quick aptamer selection in laboratories utilizing capillary electrophoresis systems (Zhuo et al., 2017).

### 3.2 Recent advancements in aptamer-based EV detection methods

Aptamers have emerged as attractive alternatives to antibodies for detecting EVs due to their high binding affinity, chemical stability, and low immunogenicity. However, various technological obstacles impede their wider clinical translation. Maintaining aptitude and affinity under physiological settings is a significant problem, as variables like pH, salt content, and nucleases in biological fluids may damage aptamers or reduce their binding efficacy (Hu and Gao, 2025).

### 3.3 Selection of aptamers for EV detection

Since its discovery, SELEX has garnered significant research interest due to its potential applications in pharmacology, medicine, and environmental analysis. While aptamers offer high affinity and specificity for diverse targets and can function under non-physiological conditions, several challenges and limitations exist. Selecting a suitable SELEX protocol requires careful consideration of factors such as RNA handling capabilities, equipment availability, desired dissociation constant (K_d_), target nature, time and cost efficiency, and potential post-SELEX modifications (Mascini and Mascini, 2009).

SELEX faces limitations due to the complexity of its processes. The immense complexity of oligonucleotide libraries necessitates amplification of functional sequences, which can introduce uncontrollable selective pressures during amplification. Additionally, the interplay between various kinetic parameters in affinity chromatography can skew selection outcomes, favoring molecules with high dissociation rates. Furthermore, when limited amounts of ligands are available, elution of functional aptamers under denaturing conditions can introduce artifacts due to matrix binding. The absence of an ideal SELEX protocol necessitates careful selection among various modifications, each with its own limitations. Cost is a significant factor, as synthesizing a massive oligonucleotide library (10^15^ sequences) requires robotic stations. Time optimization has driven the development of automated SELEX methods like the MMS chip, enabling large-scale production and reduced selection times. Aptamer affinity is another crucial consideration. While modifications have yielded aptamers with K_d_ values ranging from picomolar to millimolar, those with picomolar and nanomolar affinities are generally preferred. When time and cost constraints are paramount, SELEX protocols generating millimolar Kd aptamers may be suitable (Klug and Famulok, 1994). To overcome these issues, researchers have explored strategies such as multi-target aptamer approaches, which enhance capture specificity by simultaneously recognizing multiple surface markers on EVs. Modifications to buffer conditions, microchannel design, and surface immobilization techniques have also been used to preserve aptamer structure and enhance binding efficiency within microfluidic systems (Hu and Gao, 2025).

## 4 Aptamer-based optical biosensors for EV detection

### 4.1 Fluorescence detection

Aptamer-based fluorescence biosensors are simple to handle, sensitive, and have strong signal-to-noise ratio. These sensors depend only on fluorophores such as dyes or fluorescent nanomaterials. These types of aptasensors are operated based on three major principles, such as fluorescence signal amplification (FSA), fluorescence resonance energy transfer (FRET), and fluorescence polarization (FP) as shown in Table 3. A variety of signal amplification and fluorescence quenching materials have been used in sensor developments. Wang et al. have used DNase I enzyme-aided fluorescence signal enhancement methods using a graphene-aptamer interaction strategy for the detection of colorectal cancer exosome detection. Fluorophore-labeled CD63 and EpCAM aptamers were used for the detection of the respective biomarkers in the exosomes. Interaction of GO with the aptamers leads to quenching of aptamer fluorescence by FRET. However, in the presence of target exosomes having CD63 and EpCAM proteins, the GO surface adsorbed aptamers detached and bind to the respective protein markers. DNase I digests the ssDNA aptamer in the CD63 and EpCAM protein-aptamer complex, leaving the free exosome for the next cycle. The limit of detection for colorectal cancers (CRC) exosomes is 2.1 × 10^4^ particles/μL (Wang H. et al., 2018), demonstrating a sensitivity that is 100 times greater than that of commercial ELISA immunoassays utilizing anti-CD63 and anti-EpCAM antibodies for exosome detection in buffer (System Biosciences) (Xia et al., 2017). This underscores the advancement of a rapid and highly sensitive detection method. Usually, the LOD are obtained by comparing fluorescence emission spectra before and after exosome concentration (Wang H. et al., 2018).

**TABLE 3:** Aptamer based EV detection methods.

Category	Subcategory	Description	Advantages	Limitations	LOD	Reference
Fluorescence detection	FRET-Based Detection	GO-Based Quenching • Quench fluorophore-labeled aptamers • Restore fluorescence upon exosome binding	High sensitivity, low-cost materials, rapid detection	Potential issues with nonspecific binding or background fluorescence	2.1 × 10^4^ particles/μL	Wang et al. (2018a)
	Fluorescence Signal Amplification (FSA)	BRCA: Amplifies signal for MUC1 detection in gastric cancer exosomes	High amplification, suitable for low-abundance biomarkers	Requires additional reagents and more complex setup	4.27 × 10^4^ exosomes/mL	Huang et al. (2020b)
	Fluorescence Polarization (FP)	AFPExo Assay • Amplifies fluorescence polarization signal • Uses mass difference between aptamer and exosome	Simple, does not require multiple steps or signal amplification	Lower sensitivity compared to other techniques (e.g., FRET, FSA)	13 particles/mL	Zhu et al. (2021)
Colorimetric detection	FITC-Oxidase Mimic System	FITC oxidizes TMB under 365 nm light after binding to exosomes via cholesterol-modified ssDNA	Simple setup, naked-eye detection, light-controlled reaction	Requires UV light, potential photobleaching	1.77 × 10^5^ particles/mL	Zheng et al. (2024), Zheng et al. (2024)
	G-Quadruplex DNAzyme Inhibition	G-Rich Aptamer forms DNAzyme that • Catalyzes TMB oxidation • Disrupts structure if exosomes occur	High specificity, inversely proportional signal	Sensitive to structural conformation and competing G-rich sequences	3.94 × 10^5^ particles/mL	Kuang et al. (2022), Kuang et al. (2022)
	Anion Exchange (AE)-Fe3O4-Aptamer Colorimetric Assay	Aptamer-functionalized Fe_3_O_4_ NPs exhibit enhanced peroxidase-like activity for TMB oxidation upon binding to exosomes	Fast isolation (30 min), no need for additional enzymes	Lower sensitivity than others	3.58 × 10^6^ particles/mL	Chen et al. (2018a)
	AuNP Aggregation-Induced Color Change	Aptamer-functionalized AuNPs aggregate differently with exosomes, shifting color from red to purple/blue	Visual detection, simple chemistry, comparable to Western blot	Limited quantification, prone to nonspecific aggregation	0.7 ng/μL (EpCAM protein level)	Wang et al. (2021), Wang et al. (2021)
	Paper-Based Lipid Bilayer Magnetic Interface + HCR	Dual aptamer system isolates and amplifies signal via HCR; integrated into a paper-based chip	High sensitivity, portable format, dual-aptamer specificity	Slightly more complex setup, requires magnetic separation and paper device	5.0 EVs/μL	Ye et al. (2024), Ye et al. (2024)
	SMB + Exo-III + DNAzyme Colorimetric System	SMBs capture sEVs, release trigger for Exo-III amplification, DNAzyme catalyzes TMB oxidation for signal output	Ultra-sensitive, enzymatic amplification, effective signal generation	Multi-step process, requires Exo-III and DNAzyme design	10^2^ particles/μL	Zhang et al. (2022), Zhang et al. (2022)
SPR detection	Au@PDA NP + CD63 Aptamer	Uses aptamer-functionalized gold nanoparticles to amplify SPR signal for hepatic carcinoma exosomes	High specificity; no pre-treatment needed	Moderate sensitivity; requires nanoparticle synthesis	5.6 × 10^5^ particles/m	Liao et al. (2020), Liao et al. (2020)
	Tyramine Signal Amplification (TSA) + Molecular Aptamer Beacon (MAB)	Detects HER2-positive exosomes with G4-hemin catalysis and tyramine-coated AuNPs	Label-free; distinguishes HER2-positive exosomes	Multistep reactions; depends on catalytic efficiency	1.0 × 10^4^ particles/mL	Chen et al. (2021)
	Dual AuNP-Assisted SPR Sensor	Employs controlled aptamer hybridization and plasmonic coupling between Au film and AuNPs for high sensitivity	Ultra-sensitive; strong signal gain from nanoparticle coupling	Complex design; hybridization-dependent	5 × 10^3^ exosomes/mL	Wang et al. (2019a), Wang et al. (2019a)
SERS detection	Microfluidic platform with salt-induced AuNP aggregation	Utilizes HER2 aptamer and salt-induced AuNP aggregation to detect HER2-positive exosomes, with high sensitivity	High sensitivity; enables rapid and label-free detection	Requires microfluidic setup; limited to HER2-positive detection	4.5 log_10_ particles/mL	Ho et al. (2024)
	Multiplex detection using magnetic nanobeads and SERS probes	Gold Shell Magnetic Beads and SERS Probes that • Detect different exosomes from blood samples • Functionalized with aptamers	Allows detection of multiple exosome types; applicable to blood samples	Complex probe design; potential non-specific binding	SKBR3: 32 exosomes/μL T84: 73 exosomes/μL LNCaP: 203exosomes/μL	Wang et al. (2018a), Wang et al. (2018b)
Electrochemical detection	Nanoparticle-enabled Immunoassay	Uses nanoparticles to quantify EVs and podocin/nephrin expression on urinary EVs, varying by specific EV type	High sensitivity, ability to quantify specific biomarkers, and usefulness for diagnostic applications (e.g., preeclampsia)	Requires nanoparticle functionalization and is specific to certain biomarkers	NA	Lee et al. (2023)
	Electrochemical Aptasensor	Uses aptamers to selectively bind EVs, generating an electrochemical signal for cancer cell-derived exosome markers like CD63 and other EVs	High sensitivity, precise detection of cancer-related EVs, and miniaturizable for point-of-care use	Specific to selected biomarkers	• Total exosomes: 9.3 × 10^7^ • Cancer-derived exosomes: 7.1 × 10^8^	Kasetsirikul et al. (2022)
	Nanoarchitectonics-based Electrochemical Aptasensor	Designed for efficient exosome detection using aptamers for specific biomarkers in blood, serum, or culture media samples from cancer and infectious diseases	Highly efficient detection, suitable for monitoring disease progression	Limited to exosome detection and may require optimization for different biomarkers	NA	Javed et al. (2024)
	QCM-D Electrochemical Detection	QCM-D EV Detection • Measures binding frequency changes • Targets general exosome markers in complex fluids	High sensitivity, works well in complex biological fluids, and is suitable for clinical diagnostics	Requires specialized equipment and surface modification for different applications	NA	Suthar et al. (2023)
	Fluorescent Aptasensor-based Electrochemical Detection	Fluorescent Aptasensor and Electrochemical Detection • Precisely analyzes CD63 on exosome surfaces • Detects EVs as small as 100 nm	Dual-staining for high sensitivity, precise quantification of EVs, and suitable for clinical diagnostics	Requires a complex setup and may have potential interference from other biomolecules	NA	Du et al. (2020)
Mass-based detection	TiO2 Microsphere Method	TiO2 microspheres improve EV purity through their interaction with EV phospholipids, facilitating comprehensive protein analysis and biomarker discovery	High purity and yield of EVs, reduced contamination	Requires specialized materials and equipment	Low-abundance proteins	Santiago et al. (2024)
	Tandem Mass Tag (TMT) Proteomics	TMT measures EV proteins obtained through various isolation techniques, uncovering unique molecular profiles for biomarker investigation	Detailed proteomic analysis	Requires advanced proteomics	Varies by method	Abyadeh et al. (2024)
	Gradient Ultracentrifugation		High purity and yield of sEVs	Time-consuming, equipment needed	High sensitivity	Sharma and Dhamija (2024)
	Mass Spectrometry (MS)	MS analyzes extracellular vesicle proteins, facilitating the characterization of extracellular vesicle subtypes and potential biomarkers	Detailed EV proteome, high sensitivity	Co-isolation of contaminants	Minute quantities of proteins	Askeland et al. (2020)
	EVID-Biochip	Immunomagnetic beads and mass-based detection effectively identify L1CAM-positive EVs, which are valuable for diagnosing neurological diseases	High sensitivity (1 pg/mL), rapid detection	Requires specific antibodies	1 pg/mL	Li et al. (2024a)
	Mass Spectrometry for DNA Detection	MS isolates and detects EV DNA in serum, detecting Chagas disease early	Detects cfDNA, exovesicle DNA	Needs DNA amplification	High sensitivity for DNA	Lozano et al. (2023)
	ToF-SIMS	ToF-SIMS and machine learning detect neuroinflammation indicators in EV chemical alterations	High-resolution detection, small sample volume	Requires specialized equipment	High sensitivity for chemical variations	Bamford et al. (2023)

Abbreviations: LOD, limit of detection; FRET, Förster Resonance Energy Transfer; GO, graphene oxide; BRCA, breast cancer; MUC1, Mucin 1; AFPExo, Alpha-fetoprotein Exosome Assay; TMB, 3,3′,5,5′-Tetramethylbenzidine; G4, G-quadruplex; NPs, Nanoparticles; AuNPs, Gold Nanoparticles; HCR, hybridization chain reaction; sEVs, Small Extracellular Vesicles; SMBs, Superparamagnetic Beads; Exo-III, Exonuclease III; SPR, surface plasmon resonance; TSA, tyramine signal amplification; MAB, molecular aptamer beacon; SERS, surface enhanced raman spectroscopy; HER2, Human Epidermal Growth Factor Receptor 2; qPCR, quantitative polymerase chain reaction; CD63, Cluster of Differentiation 63; QCM-D, quartz crystal microbalance with dissipation; MS, mass spectrometry; TiO2, titanium dioxide; TMT, tandem mass tag; EV, extracellular vesicle; L1CAM, L1 cell adhesion molecule; cfDNA, Cell-Free DNA; ToF-SIMS, Time-of-Flight Secondary Ion Mass Spectrometry.

Furthermore, Early-stage detection of and classification of cancer has been achieved using thermophoretic profiling of extracellular vesicle surface proteins. Interestingly, seven different fluorescence-labelled aptamers have been used for the classification of 6 different types of cancer stages I–IV from 102 patient samples. This assay detects stage I cancer with 95% sensitivity (95% confidence interval), 100% specificity, and 68% accuracy (Liu et al., 2019).

Huang et al. pioneered a fluorescence amplification method for the sensitive detection of gastric cancer exosome biomarkers. Using an aptamer-specific exosome target, a simple fluorescence aptasensor for gastric cancer exosome detection based on branched rolling circle amplification (BRCA). Mucin 1 (MUC1), a cell surface glycoprotein, was used as an exosome target membrane protein. After capturing the exosome, the aptamer-exosome complex was broken down by high temperature, and the partial complementary to aptamer padlock probe triggered the amplification. The resulting dsDNA was quantified using SYBR GREEN fluorescence. A high specificity with a low LOD of 4.27 × 10^4^ exosomes/mL was achieved (Huang R. et al., 2020). CD63, an exosomes transmembrane protein-specific aptamer-based fluorescence assay was used for the qualitative detection of exosomes. Aptamer conjugated magnetic beads were hybridized Cy3 labelled partial complementary to the aptamer sequences. When the exosome sample are introduced into the system, the fluorescently labeled short sequence is released into the solution by competitive binding of CD63 protein. The quantity of exosomes present in the sample was correlated with the fluorescence signal enhancement. This method demonstrated the LOD of 1.0 × 10^5^ particles/μL under optimal conditions (Yu et al., 2019).

Recent advancements in the field of exosome detection have led to the development of innovative nanosensors. One such innovation is the homogenous magneto-fluorescent exosome (hMFEX) nanosensor, which facilitates rapid onsite detection of tumor-derived exosomes. This sensor operates through the immune magnetic capture of exosomes, triggered by the assembly of DNA three-way junctions in a solution containing an aggregation-induced emission probe and graphene oxide (GO). Notably, the fluorescence enhancement observed with the hMFEX nanosensor is directly correlated with the presence of exosomes in the solution, allowing for detection of as low as 6.56 × 10^4^ particles/μL (Li et al., 2020). In a separate approach, Dong et al. demonstrated effective separation and quantification of EVs using the integrated ExoID-Chip, which incorporates a photonic crystal nanostructure, a double-filtration unit, and ultrasensitive nanofiltration membranes to isolate EVs sized between 20 and 200 nm through size exclusion. The EVs are enriched on the membrane, and the excess amount of CD63 labeled aptamers was exploited for the quantitative detection of EVs with CD63 biomarkers using competitive immunoassay (Dong et al., 2019). Therefore, this approach can effectively differentiate between breast cancer patients and healthy individuals.

Another highly sophisticated method employs luminescence techniques to measure exosomes via energy transfer mechanisms, offering exceptional sensitivity and specificity. Luminescence resonance energy transfer (LRET) between rare-earth-doped upconversion nanoparticles (UCNPs) donor and tetramethyl rhodamine (TAMRA) acceptor was used for the highly sensitive detection of detection of exosomes. Epithelial cell adhesion molecule (EpCAM) is a highly expressed surface protein of exosomes. EpCAM-specific aptamer was split into two ssDNA strands labeled with UCNPs and TAMRA, respectively. In the presence of a target exosome, the two strands join together and the donor and acceptor are close to each other and favor the energy transfer between UCNPs and TAMRA. The ssDNA-UCNPs-ssDNA-TAMRA-exosome complex was excited by near-infrared light at 980 nm, due to LRET, the yellow fluorescence of TAMRA at 585 nm was observed. The fluorescence intensity at 585 nm is correlated with the quantity of exosomes in the sample. The LOD of this method was 80 particles/μL (Wang Y. et al., 2019). This strategy underscores the potential of LRET-based aptasensors for precise exosome quantification, although further validation in complex biological samples is necessary for clinical translation.

Other subsequent studies have explored alternative donor–acceptor pairs and aptamer configurations to enhance sensitivity and adaptability for exosome detection in various formats. Aptasensors have shown great promise for exosome detection by leveraging diverse mechanisms such as LRET and structure-switching strategies. In one example, Chen et al. designed a dual aptamer-based LRET sensor using upconversion nanoparticles (UCNPs) and gold nanorods (Au NRs) as donor–acceptor pairs (Chen X. et al., 2018). One aptamer was immobilized on filter paper coated with UCNPs, while the other was labeled with Au NRs. Upon binding the CD63 protein on exosomes, the aptamers brought UCNPs and Au NRs into close proximity, leading to LRET-mediated quenching of luminescence. The quenching efficiency correlated linearly with exosome concentration, achieving a detection limit of 1.1 × 10^3^ particles/μL (Chen X. et al., 2018).

In another approach, Chen and co-workers developed target molecule-activatable structure-switching aptamer platform for detecting exosomes. Tyrosine kinase-7 (PTK7) specific aptamer sgc8 was used as a model. In this model, the sequences are used a recognition domain and rest of the sequences are used as trigger domain for the displacement reaction. N-methylmesoporphyrin IX (NMM) is used a fluorescent probe, which can intercalate into the G quadruplex structure and generate a strong fluorescence. In the absence of a target, the G-rich DNA sequences were duplexed with complementary DNA, which hindered the G-quadruplex structure formation. In contrast, in the presence of target exosomes, sgc8 aptamer bound to PTK7 by structure structure-switching mechanism of the target binding aptamer, recognition domain. On the other the cDNA displacement in the trigger domain forms a G-quadruplex, which allows NMM to form an NMM-G-quadruplex complex, thereby increasing the fluorescence signal. A linear relationship between the exosome quantity in CCRF-CEM cells with the fluorescence signal was established and the LOD of this method was3.4 × 10^5^ particles/µL (Chen et al., 2020). Efficient detection of breast cancer cell-derived exosomes was demonstrated using the bicyclic capture probe, which consists of an HER2-specific aptamer and G4-hemin catalytic enzyme. The over-expressed HER2 protein SK-BR3-derived exosomes are exploited as a target in the exosome detection methodology. The aptamer bicyclic capture probe binds to the HER2 exosome membrane protein and the G4 hemin sequence was exposed, which further interacts with hemin and the G4-hemin catalytic enzyme was generated. Finally, tyramine initiates reg catalysis of G4-hemin and produces the fluorescence signal. The linear detection range from 2.5 × 10^5^ to 1.00 × 10^7^ breast cancer cell-derived exosome particles/mL was tested and the LOD was as low as 0.54 × 10^5^ particles/mL (Chen et al., 2022).

**FIGURE 6:**
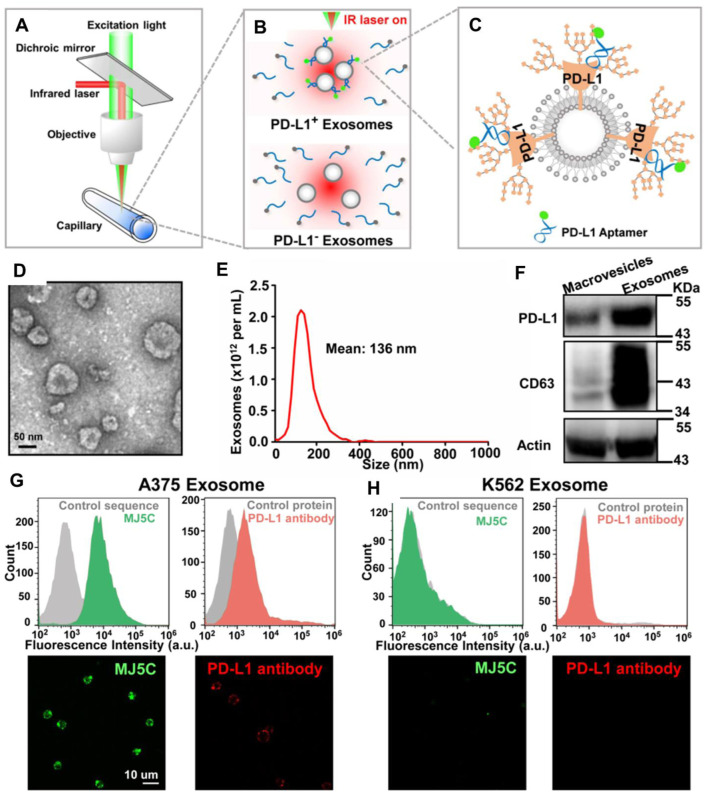
**(A–C)** Working principle of homogeneous, low-volume, efficient, and sensitive exosomal PD-L1 (HOLMES-ExoPD-L1) quantitation method. **(D)** Characterization of vesicles surrounded by a lipid layer, appearing as the characteristic of exosomes using TEM. **(E)** Characterization of purified exosomes using nanoparticle tracking analysis. **(F)** Western blotting analysis confirmed the expression of PD-L1 and CD63. **(G,H)** flow cytometry assay and confocal images demonstrate the binding performance of the MJ5C aptamer and PD-L1 antibody to PD-L1 positive exosome conjugated beads **(F)** and negative exosome conjugated beads **(G)**. Reproduced from ref Huang M. et al. (2020) with copyright permission.

To enable accurate and portable tumor exosome detection, Chen et al. (2024) developed a dual-mode lateral flow assay based on manganese dioxide (MnO_2_) and aptamer-functionalized fluorescent microspheres. Specifically, this colorimetric and fluorescence-based sensor utilizes aptamer-functionalized fluorescent microspheres (FMs-aptamer) as donors and MnO_2_ as an acceptor for detecting MCF-7-derived exosomes. The test line on the strip contains FAM-labeled MUC1 aptamer, and after sample migration, MnO_2_ is added as a fluorescence quenching agent. In the presence of target exosomes, the aptamer captures the exosomes, preventing close contact between FAM and MnO_2_. As a result, fluorescence resonance energy transfer (FRET) does not occur, and a strong FAM fluorescence signal is observed. Conversely, in the absence of target exosomes, the FAM and MnO_2_ come into proximity, enabling FRET and quenching the FAM fluorescence. Additionally, the brown-colored test line—indicating aggregated MnO_2_—can be visually detected. This method achieves a low detection limit of 2.5 × 10^3^ particles/mL and is well-suited for point-of-care testing without the need for complex sample pretreatment (Chen et al., 2024). In a related approach, Guo et al. (2023) introduced a dual-aptamer recognition system targeting two different EV membrane proteins—CD63 and PTK7. Here, UiO-66-NH_2_ was functionalized with one aptamer to isolate EVs, while a second aptamer triggered rolling circle amplification (RCA) for signal amplification. The RCA-generated product contained G-quadruplex (G4) antisense sequences capable of binding thiosemicarbazone T (ThT), resulting in fluorescence emission. Importantly, the fluorescence intensity directly correlated with the EV concentration, enabling quantitative detection. The method successfully measured EV concentrations ranging from 5 × 10^4^ to 1 × 10^7^ particles/μL, with a detection limit of 2.2 × 10^4^ particles/μL (Guo et al., 2023). The working principle of the Holmed-ExoPD-L1 platform, which is illustrated in Figure 6, further exemplifies the growing innovation in aptamer-based exosome detection systems (Huang M. et al., 2020).

Aptamer-functionalized magnetic nanoparticle platforms have developed into effective instruments for the isolation and detection of exosomes, demonstrating high sensitivity and specificity for diseases. To illustrate, aptamer-based lung cancer exosome detection using epithelial malignant tumor marker EpCAM as a target. For exosome isolation, an aptamer-functionalized FRET magnetic nanoparticle was designed. Anti-EpCAM aptamer and its complementary sequences were used to make a bridge between the QDs and the Au for an efficient FRET pair. In the absence of a target exosome, the FRET occurs between QDs and AuNPs. However, in the presence of an exosome, the partial complementary sequence dissociates, and EpCAM on the exosome surface binds to the aptamer and releases the Au-coupled complementary. This process leads to the separation of FRET pair and no FRET between QDs and AuNPs. Therefore, a highly intense fluorescence signal was observed. The concentration of the exosome is correlated with an increase in the fluorescence signal of QDS. The exosomes derived from A549 cell lines in the range between 5 × 10^2^ and 5 × 10^9^ particles/mL were measured. The method shows the LOD of 13 particles/mL (Zhu et al., 2021).

**FIGURE 7:**
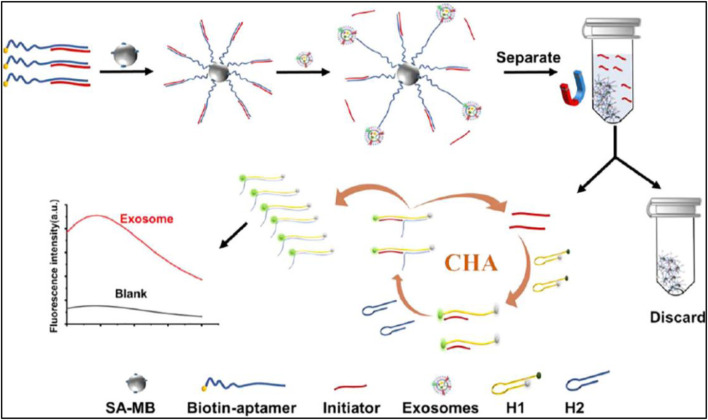
The working principle of the aptamer-initiated CHA (AICHA) signal amplification method for exosome detection is outlined. H1 was tagged with a FAM fluorophore and a BHQ2 quencher. SA-MB stands for streptavidin-modified magnetic beads, and Biotin-aptamer represents the biotin-labeled aptamer. Reproduced from ref Zhou et al. (2022), with copyright permission.

Another example have been developed by Wang et al. where magnetic nanoparticles were conjugated with Anti-CD63 aptamer for the isolation of exosomes for Alzheimer’s disease detection. To facilitate the binding affinity of mesoporous Fe_3_O_4_ nanoparticles, gold nanoparticles were deposited on the surface. The thiolated Anti-CD63 aptamer was conjugated on the surface of Fe_3_O_4_@Au via gold–sulfur bonds. The conjugated aptamer recognizes Alzheimer’s disease-specific exosome biomarkers through the CD63 surface protein. Finally, 1.0 M NaCl solution was used to elute the bound exosomes. The characterization and quantification have been done by further downstream analysis (Wang et al., 2023). Similarly, a fluorescence assay based on aptamer-initiated catalytic hairpin assembly (AICHA) was designed for detecting cancer-cell-derived exosomes. The protein-specific biotin-modified aptamer conjugated with streptavidin-modified magnetic beads (SA-MB). A partial complementary sequence of the aptamer is reused as an initiator. In the presence of MCF-7 cell-derived exosomes, the aptamer binds to the exosome surface protein and releases the short initiators into the solution. These initiators hybridized with reported DNA probes having FAM and BHQ2 FRET pair, and another hairpin DNA will hybridize and form a perfect duplex, as a result, the fluorescence of the quenched FAM recovered and showed bright fluorescence. The method was tested in the range of 8.4 particles/μL to 8.4 × 10^5^ particles/μL and the LOD of this method was 0.5 particles/μL (Zhou et al., 2022) in Figure 7. Therefore, this aptamer-triggered catalytic system presents a remarkably sensitive and specific fluorescence-based method for exosome detection, underscoring its promisefor early cancer diagnostics.

Fluorescence Polarization/Fluorescence Anisotropy (FP/FA) is a sensitive technique that measures the rotational diffusion of a fluorescent probe. When a fluorescent probe is attached to an aptamer, changes in the aptamer’s conformation upon target binding affect the rotational speed of the probe, resulting in alterations in FP/FA values. This principle allows for the quantitative detection of target molecules with high sensitivity and specificity (Jameson and Ross, 2010; Zhao et al., 2020). Fluorescence aptasensors are primarily categorized into labeled and label-free types, each capable of indicating target binding through either signal enhancement (turn-on) or reduction (turn-off) (Zhao X. et al., 2021). Therefore, an application to this principle is a separation-free, amplification-free aptamer-based fluorescence polarization assay that was developed for the sensitive quantification of exosomes from human plasma (AFPExo assay). The large mass/volume of the exosome was exploited for the fluorescence polarization amplification. The exosome surface protein (CD63) was recognized by the high-affinity low-molecular-weight aptamer. The molecular mass of the aptamer is about 10 kDa, whereas the molecular mass exosome is 3.3 × 10^4^ kDa. Therefore, the dye-labeled aptamer leads to a significant change in the molecular mass of exosome-aptamer complex that reflects in the huge variation in the fluorescence polarization signal. This assay was tested with the exosome concentration range of 5 × 10^2^ to 5 × 10 particles/μL, with a LOD of 500 particle/μL of cell line derived exosomes (Zhang et al., 2019).

In addition to polarization-based strategies, ratiometric fluorescence methods also enable accurate exosome quantification by using dual-dye systems that respond differentially to exosome membrane interactions. In this method, Li et al. quantified exosomes by the total Membrane Lipid Assay (MLA), in which two different dyes were used. One of the dyes is an exosome membrane-specific dye that was non-fluorescent in buffer while it bound with the membrane phospholipid bimolecular layer, it emits a strong fluorescence, The other dye does not influenced by the exosome. Therefore, the farmer dye is was used as monitoring the exosome and the later dye was used as an internal reference. The fluorescence intensity ratio of the dyes was exploited for the quantification of exosomes present in the sample. The LOD of this method was 0.342 ng/μL (MLA total membrane lipid content) (Li et al., 2018). The MLA’s low LOD outperforms previous label-free fluorescence assays, indicating its potential for clinical-grade exosome quantification. (Huang et al., 2025).

**FIGURE 8:**
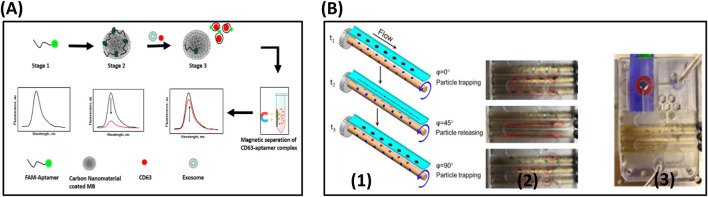
**(A)** Schematic diagram outlines the fluorescence switching mechanism in the fluorescence assay for exosome CD63 detection, based on carbon nanomaterial-coated magnetic nanobeads. Stage 1: The free aptamer emits a strong fluorescence signal. Stage 2: Fluorescence is quenched due to the interaction with the carbon nanomaterial on the magnetic nanobeads. Stage 3: Upon introduction of the target sample, the aptamer detaches from the carbon surface, binds to the target with high affinity, and results in increased fluorescence intensity. **(B)** (1) The trapping and releasing mechanism is depicted in the schematic. Magnetic particles are trapped when the magnet pole faces the channel (φ = 0°), and released when it moves out of the channel’s plane (φ = 90°). (2) A series of images capture the alternating trapping and releasing process as the rMAS rotates, as shown in the red circles. (3) The final trapping of the particles in the reservoir (Ⓡ) is achieved using a stationary magnet (M), indicated by the red circle. Reproduced from ref Chinnappan et al. (2023b) with copyright permission.

Kalimuthu et al. have also demonstrated the accurate and quantitative detection of EV by the fluorescence polarization method. The lipophilic fluorescein probe, 5-dodecanoylamino fluorescein (C12-FAM) is made up of an aliphatic, alkyl tail was used as the FP probe. Due to the lipophilic nature of C12-FAM, it would be inserted into EVs and a significant increase in the volume of probe leads to a remarkable change in FP signal by restricted rotations. This method can detect as low as 17.5 × 10^5^ EVs/μL (Kalimuthu et al., 2019). Labeled aptasensors often employ FRET for turn-on signals, where fluorophore quenching is reversed upon target binding and conformational change. Anti-CD63 aptamer was used for the isolation of colorectal tumor-induced exosomes. In which, the fluorescence of FAM-labelled aptamer was adsorbed on the nano material-coated magnetic beads was quenched by FRET processes in the absence of Exosomes. However, in the presence of an exosome, the aptamer detaches from the surface of the beads and recovers the fluorescence as illustrated in Figure 8. Aptamer-conjugated magnetic beads were used for concentrating the exosomes released from the tumor cells. A trap and release mechanism, where the magnetic particles are trapped when the magnetic pole is pointing at the channel and released when the pole is out of the face as shown in the figure. Finally, the exosomes are captured on the surface of the beads by an anti-CD63 aptamer. At the end of the flow process, a magnet is placed under the channel and collects the magnetic beads separated the exosomes by a change in the pH of the medium. The LOD of this method was 1.45 × 10^3^ particles/mL (Chinnappan et al., 2023b) described in Figure 8.

Alternatively, sandwich assays using aptamer pairs or aptamer-antibody combinations generate turn-on signals. Turn-off labeled aptasensors typically involve fluorophore-quencher pairs brought into proximity by target-induced conformational changes (Zhao et al., 2018). Label-free aptasensors can achieve turn-on signals through fluorophore displacement upon target binding or by activating specific sequences like G-quadruplexes that bind fluorescent dyes. Additionally, fluorophore displacement can induce turn-off signals in label-free aptasensors (Zhou et al., 2019).

Another novel fluorescence aptasensor utilizing high-resolution flow cytometry (FCM) has been developed for the precise quantitative detection of nano-sized membrane vesicles, specifically exosomes (EVs). The EVs of 100 nm sizes were quantitatively analyzed using a dual staining procedure by CD63 targeting aptamers and cytoplasmic dye. The nano-sized EVs derived from bone marrow mesenchymal stem cells, human neural stem cells, and human corneal epithelial cells were used for the analysis. The quantity of EVs varies in the range of 6.79 × 10^6^ particles/mL to 2.08 × 10^8^ particles/mL (Du et al., 2020).

### 4.2 Colorimetric detection

A colorimetric aptasensor employing the light-stimulated oxidase-mimicking activity of fluorescein isothiocyanate (FITC) enabled the quantitative detection of ovarian cancer (OC) exosomes. This involved using an EpCAM aptamer to capture the OC exosomes. A ssDNA was modified with Cholesterol and fluorescein (FITC) on both ends. The hydrophobic cholesterol attached with the exosome through hydrophobic interaction, and the other end with FITC was used to oxidize 3,30,5,50-tetramethylbenzidine (TMB) under 365 nm irradiation by LED light source temporally controllable manner under mild conditions. As a results, the TMB is oxidized (TMBox) and the solution color changes to blue from colorless. The qualitative detection of exosomes was achieved by naked-eye observation. The quantitative detection was done using the UV-VIS spectroscopic method. The concentration of OC exosomes was tested in the linear range of 2 × 10^5^ to 100 × 10^5^ particles/mL and the LOD was 1.77 × 10^5^ particles/mL (Zheng et al., 2024).

Moreover, among colorimetric strategies for exosome detection, DNAzyme-based aptasensors have emerged as promising tools due to their simplicity and visual readout. For instance, Kuang et al. reported a highly specific and sensitive aptasensor for the detection of exosomes using EpCAM aptamer as a capturing element. The wild-type Guanine-rich EpCAM aptamer formed G quadruplex and it forms hemin/G4 complex DNAzyme. It catalyzed the TMB in the presence of H_2_O_2_ and produced blue-colored oxTMB. However, in the presence of the exosome target, the DNAzyme structure was disturbed and the catalytic activity was inhibited. The absorbance of the reacting solution was inversely correlated to the concentration of exosomes present in the sample. The concentration of exosomes in the range of 10^6^–10^8^ particles/mL was measured and the calculated LOD was 3.94 × 10^5^ particles/mL (Kuang et al., 2022).

**FIGURE 9:**
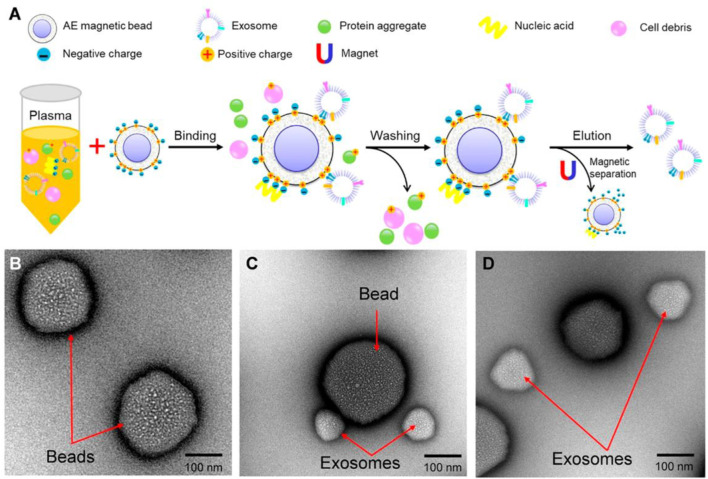
Anion exchange-based isolation method of exosomes. **(A)** Schematic representation of AE-based isolation of exosomes. **(B)** Characterization of beads using Transmission electron microscopy (TEM). **(C)** Characterization of beads after capture of exosomes using TEM. **(D)** Characterization of AE magnetic beads and exosomes after elusion using TEM. Reproduced from ref Chen J et al. (2018) with copyright permission.

In contrast to enzyme-mimicking aptamer structures, Anion exchange (AE) based isolation of exosome from plasma was demonstrated within 30 min with high purity. The AE magnetic beads were functionalized with aptamer-modified Fe_3_O_4_ nanoparticles for specific binding and isolation of PCa exosomes. The AE magnetic beads. Aptamer-modified Fe_3_O_4_ NPs are used for the isolation of PCa exosome. Fe_3_O_4_ NPs have a weak peroxidase-like activity; however, the aptamer modification facilitates the peroxidase-like activity of Fe_3_O_4_ NPs and catalyzes TMB and changes the color to blue. This method facilitates rapid detection of exosomes, estimating PCa exosomes within a linear range of 0.4 × 10^8^ to 6.0 × 10^8^ particles/mL, with a LOD of 3.58 × 10^6^ particles/mL Figure 9 (Chen J. et al., 2018).

Furthermore, a third method relied on nanoparticle (NP) aggregation to produce a visible color shift was the aggregation-induced color change in the AuNPs usage for the detection of exosomes. Three different membrane target proteins are used for detection purposes. The Au nanoparticles were modified with thiolated aptamers. Upon addition of Au growth chemicals, mixture of potassium tetrachloroaurate and hydroxylamine hydrochloride, the charged functional group in the aptamer binds to Au^3+^ and facilitates the crystal growth, and the color changes from light red to deep red. However, in the presence of an exosome target, the AuNps binds to the surface target proteins of the exosomes and changes the color from red to purple and blue. The EVs derived from (MCF-7 and MDA-MB-231) were comparable with Western blotting results. This method demonstrated the LOD of 0.7 ng/μL EpCAM based on the MCF-7 EVs (Wang et al., 2021).

An alternative, unique method is the development of a paper-based lipid bilayer magnetic interface for the ultrasensitive and quantitative assessment of tumor-derived extracellular vesicles (T-EVs) that express PD-L1. In this method, the lipid bilayer magnetic interface served as an isolation and enrichment carrier integrated with a hybridization chain reaction (HCR) as a signal amplification in the sensing paper device. The magnetic beads were modified with a lipid bilayer and cholesterol-modified EpCAm aptamer was used for the construction of high-performance isolation of T-Evs. PD-L1 aptamer was used to initiate the HCR of EVs to amplify the signal. This dual aptamer paper-based biosensor showed an LOD of 5.0 EVs/μL (Ye et al., 2024).

Streptavidin magnetic beads (SMBs)-capture probe-assisted sEVs identification is another example. An Exo-III-assisted signal amplification system was built to find small extracellular vesicles (sEVs) using a colorimetric method. This sensing method consists of target sEVs recognition-mediated liberation of trigger sequence, signal amplification by Exo-III, and color development or signal output by DNAzyme catalysis. This colorimetric method exhibits a detection range of 10^2^ to 10^6^ particles/L (Zhang et al., 2022).

### 4.3 SPR (surface plasmon resonance) detection of exosomes

Although multiple quantitative protein labeling strategies have been developed for biomolecular targeting, their potential binding affinity is greatly impeded by steric hindrances and alterations in morphology. A bridge to mend that gap arose by refining detection utilizing more sensitive, label-free methods, exemplified by optical biosensors (Nguyen et al., 2015). SPR, the most widely used optical biosensor, is capable of exhibiting its properties of biomaterial distinction through measurement of the corresponding refractive angle of incidence index changes. The detection process is kickstarted through emitted photons cast onto a thin metal surface, usually gold, which has the biomaterial of interest placed atop it. At a specific refractive angle, some photons from the light source are able to penetrate through the metal and charge its surface electrons, now called “plasmons,” allowing them to vibrate. This vibration propagates parallel to the metal’s surface, generating a pattern of oscillation detected by the target biomolecule. With even the slightest change of the refractive angle, a plasmon would not be formed, and the target biomolecule would not be detected. Thus, an SPR sensor is realized through the refractive index changes created by the analyte adherent to the detector surface (Nguyen et al., 2015; Ji et al., 2022). The analyte detected by SPR may range from large molecule carbohydrates and proteins all the way down to single-particle extracellular vesicles (EVs) (Nguyen et al., 2015; Yang et al., 2020). The wide-field detection of SPR allows high-quality recognition, quantification, and sizing of non-biological and biological nano-particles, including extracellular vesicles (Sharar et al., 2023). Unfortunately, despite rapid advancements, SPR is currently noted to be limited due to its inability to detect smaller concentrations, measurement of ensembles of EVs rather than their antigens, and overall sensor degeneration (Gool et al., 2017; Rikkert et al., 2020).

**FIGURE 10:**
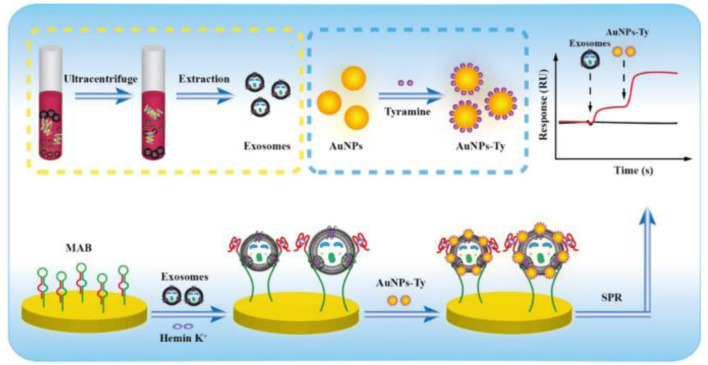
Diagrammatic representation of HER2-positive exosome detection using enhanced TSA facilitated by target-driven MAB conversion. Reproduced from ref Chen et al. (2021), with copyright permission under the terms of the CC-BY-NC-ND 4.0 license.

SPR-based detection of exosomes was demonstrated by aptamer recognition and polydopamine-functionalized gold nanoparticles (Au@PDA NP) enabled signal amplification in Figure 10 (Chen et al., 2021). The hepatic carcinoma-derived exosomes were captured by aptamer ZY-sls that were complementary to the tetrahedron probes (DTPs). The other signal amplification part consists of (Au@PDA NP) linked with CD63 aptamer. The CD63 aptamer recognizes the CD63 surface protein and enhances the SPR signal. This assay accurately distinguishes SMMC-7721 exosomes from other exosomes. The LOD of this method was found to be 5.6 × 10^5^ particles/mL without any pre-sample treatment (Liao et al., 2020). A label-free SPR biosensor was constructed for highly sensitive detection of HER2-positive exosomes based on reformative tyramine signal amplification (TSA) integrated with a molecular aptamer beacon (MAB). The MAB immobilized sensor chip captured the exosomes, enabling the G-quadruplex DNA (G4 DNA) to form peroxidase-like G4-hemin. The G4-hemin catalyzes the tyramine-coated gold nanoparticles (AuNPs-Ty) on the exosome surface in the presence of H_2_O_2_ and enhances the SPR signal. This method was tested in the wide range from 1.0× 10^4^ to 1.0× 10^7^ particles/mL. This method can accurately distinguish the HER2 Positive cancer patients from healthy individuals (Chen et al., 2021). Dual gold nanoparticle (AuNP)-assisted signal amplified SPR aptasensor was demonstrated for the sensitive detection of cancerous exosomes. The controlled hybridization of aptamer and their partial complementary leads to the attachment of AuNps forms electronic coupling between the Au film and AuNPs and coupling effects in plasmonic nanostructures resulting in the dual nanoparticle amplification. This highly sensitive method detect the exosome as low as 5 × 10^3^ exosomes/mL (Wang Q. et al., 2019).

### 4.4 SERS (surface-enhanced Raman spectroscopy) detection

As a powerful optic sensor, Surface-Enhanced Raman Scattering (SERS) technology provides highly sensitive and specific detection of various biomolecules through employing Raman scattering. (Yang et al., 2024). Raman scattering refers to the process whereby the frequency of inelastic light changes as it passes through a specific biological molecule, reflecting its properties. However, due to the paucity of approximately one out of 10^8^ photons spontaneously undergoing Raman scattering, the size, arrangement, and shape of the substrate are of crucial importance to improving the detection capacity of SERS (Wu et al., 2024a). The recent review on the detection of cancer cell-derived exosomes by SERS discussed in detail. They have summarized the SERS besed exosome detection and combination of SERS with other methodologies for the quantitative diagnosis of cancers by exosomes as biomarkers (Li et al., 2022). A droplet microfluidic platform integrated with a surface-enhanced Raman spectroscopy (SERS) was used for the rapid and quantitative detection of HER2-positive exosomes derived from cancer cells using aptamer as a recognition element. An on-chip salt-induced AuNps aggregation processes by HER2 aptamer in the presence of HER2-positive exosomes that induces the hot spot-based SERS signal amplification. This method detected as low as 4.5 log_10_ particles/mL (Ho et al., 2024). Simultaneous multiple detection of exosomes screening method was achieved using magnetic substrates and SERS probes. The gold shell magnetic nanobeads capturing substrates modified by aptamers capture the variety of exosome types recognized by CD63 surface protein aptamer. In addition, the SERS probes are made of gold nanoparticles decorated with a Raman reporter and the target-specific aptamer. Three different SERS probes were used to capture the exosomes. In the presence of the target exosome, the apta-immunocomplex formed and the other non-specific probe remained in the suspension. This method was employed to detect exosomes directly from the blood sample, which is a promising tool for cancer screening based on the exosomes (Wang Z. et al., 2018).

## 5 Electrochemical detection

Electrochemical detection is an analytical technique that measures the electrical properties of a system to indicate the presence and concentration of charged chemical substances (Ivaska, 2008). This method typically involves an electrochemical cell comprising three main components: a working electrode, a reference electrode, and a counter electrode (Giagkoulovits et al., 2018; Pozo-Ayuso et al., 2020). When a potential is applied to the electrodes, redox reactions occur, leading to a measurable electrical signal, which changes in current or potential corresponding to the analyte’s concentration (O’Brien et al., 2021).

The electrode surface is typically functionalized with the recognition element, which specifically binds to the target analyte such as a protein or nucleic acid (Bazzana et al., 2023). When the target analyte binds to the recognition element, it induces an electrochemical reaction that generates a measurable signal, usually in the form of current, voltage, or impedance (Wang, 2022).

Electrochemical detection in extracellular vesicle (EV) biosensors involves several key steps and components:

1. Electrode Preparation: The electrode is functionalized with a recognition element (e.g., antibodies, aptamers) that specifically binds to target molecules present on the EV surface. This functionalization ensures selectivity towards the desired EV markers.
2. Binding of EVs: When a biological sample containing EVs is added, the target EVs bind to the recognition elements on the electrode surface. This binding initiates the electrochemical detection process.
3. Electrochemical Transduction: The binding of EVs to the electrode alters the electrochemical environment by changing parameters such change of electrolyte, pH, temperature, etc. These changes are detected by measuring values such as current, voltage, or impedance. Common electrochemical techniques include amperometry (measuring current at a fixed voltage), potentiometry (measuring voltage changes), and electrochemical impedance spectroscopy (measuring impedance over a range of frequencies).
4. Signal Generation and Analysis: The binding of EVs causes a change in the electrochemical signal, which can be quantitatively measured. The magnitude of the signal change is proportional to the concentration of the target EVs, allowing for quantitative analysis. This signal can be amplified using various methods to enhance sensitivity (Maity and Sahu, 2022; Brinda et al., 2023).

One significant benefit of electrochemical detection is its high sensitivity, allowing EVs to be detected at very low concentrations, which is critical for early disease diagnosis and monitoring. For instance, the iPEX system can detect EVs at concentrations as low as 500 EVs/mL without secondary labeling (Kilic et al., 2022). Electrochemical detection also provides rapid results, with some methods enabling fast sensor preparation and quick measurements, which is advantageous for clinical applications (Wang, 2022). Additionally, electrochemical techniques can be precise, as they can be designed to target specific biomarkers on EVs, enhancing the accuracy of diagnostics (Liu et al., 2023). These methods are also relatively cost-effective and can be easily miniaturized, making them suitable for point-of-care testing and widespread clinical use (Dias et al., 2022).

In the context of extracellular vesicle biosensors, electrochemical detection is applied in various ways. For instance, in a study by Lee et al. (2023), a nanoparticle-enabled immunoassay integrated with an electrochemical plate was developed to quantify podocin and nephrin expressions on urinary EVs, helping to diagnose preeclampsia. Electrochemical detection is also being used for the detection and analysis of cancer-related EVs. For example, Serrano et al. (2022) used an electrochemical biosensor to detect miRNA 21-5p in human serum using screen-printed carbon electrodes coated with gold nanoparticles, allowing it to achieve a low detection limit, which implies good potential for early cancer detection. Another example is the study by Javed et al. (2024) which used a nanoarchitectonics-based electrochemical aptasensor designed for highly efficient detection of exosomes to diagnose infectious diseases and monitor disease progression.

Recent reports highlight various advancements in the isolation and detection of EVs using electrochemical methods. These methods improve sensitivity, specificity, and practicality for clinical applications. Some notable ones include (Boulestreau et al., 2024)’s study investigating different methods for isolating salivary EVs, including co-precipitation, immuno-affinity, and ultracentrifugation. They found that co-precipitation was an efficient, cost-effective method that maintained the integrity of EVs, making it suitable for clinical applications. Similarly, another instance is Koch et al. (2024) who used anion exchange chromatography (AEX) for isolating EVs. They showed that AEX could efficiently separate EVs based on surface charge interactions, proving the method to be scalable and cost-effective for EV isolation. This approach also maintained the integrity of key EV surface markers while also giving insights on the size distribution and purity of the isolated EVs. Likewise, Kasetsirikul et al. (2022) developed a low-cost electrochemical paper-based analytical device to quantify both total bulk and cancer cell-derived exosomes in cell culture media. This device employs a sandwich immune assay design, where exosomes are initially captured using electrode-bound generic antibodies (CD9) and subsequently detected via ovarian cancer-specific CA125 antibodies. The device demonstrated a detection limit of 9.3 × 10^7^ exosomes per mL for total exosomes and 7.1 × 10^8^ exosomes per mL for ovarian cancer cell-derived exosomes. As another illustration, Mathew et al. (2020) developed an electrochemical sensing scheme for tumor-derived EV (tdEV) detection using nano interdigitated electrodes. This approach achieved high sensitivity and specificity for tdEV detection in blood, with a detection limit as low as 5 tdEVs/µL, making it suitable for point-of-care cancer diagnostics. To give another example, Suthar et al. (2023) used the sensitivity of quartz crystal microbalance with a dissipation monitoring (QCM-D), an electrochemical detection method, to detect EVs by creating a 2D gold nanostructured arrays on the sensor’s surface. Using block copolymer self-assembly, they increased the surface area for EV capture, improving antibody binding and reducing steric hindrance. This led to a 4-fold sensitivity increase despite the reduced binding area. The QCM-D sensor showed high sensitivity and specificity in detecting EVs in complex fluids like urine, plasma, and saliva, indicating the potential of nanostructured surfaces for clinical diagnostics. Likewise, Du et al. (2020) developed a fluorescent aptasensor-based method for quantitative analysis of nano-sized extracellular vesicles (EVs) using high-resolution flow cytometry. The method utilized aptamers to target the CD63 protein on EV surfaces and incorporated electrochemical detection by measuring changes in electrical signals upon aptamer-EV binding. This dual-staining approach allowed precise differentiation and quantification of EVs with high sensitivity, detecting EVs as small as 100 nm. The technique demonstrated significant potential for accurate EV analysis in diverse biological samples and clinical diagnostics. Ekwujuru et al. (2023) reviewed the development of electrochemical (EC) and photoelectrochemical (PEC) biosensors for detecting ovarian cancer biomarkers. The study highlighted PEC biosensors, which combine photoelectrochemical and electrochemical principles to enhance sensitivity, offering rapid testing, low cost, and potential for miniaturization. These biosensors use light to excite photoactive materials, generating a measurable electrochemical signal upon interaction with specific biomarkers. The research emphasized the effectiveness of these biosensors in accurately detecting ovarian cancer markers, providing a promising tool for early diagnosis and monitoring. Li M. et al. (2021) utilized electrochemical detection for microRNA by integrating DNA walkers and hyperbranched hybridization chain reaction (HCR) with DNAzyme signal amplification. The DNA walkers, activated by Mg^2+−^dependent DNAzymes, initiated the HCR process, producing numerous electroactive sites. These sites formed hemin/G-quadruplex DNAzymes that catalyzed H_2_O_2_ decomposition, significantly amplifying the electrochemical signal. This method enabled highly sensitive and selective detection of microRNA-141, demonstrating its potential for assays in complex biological samples.

Another example includes an electrochemical biosensor developed by Arul et al. for detecting superoxide and nitric oxide anions, using a dendritic silver-organic framework (Ag-MOF) nanozyme that mimics enzyme properties with improved stability. Synthesized through a solvothermal process, the Ag-MOF with polymeric composites enhanced conductivity and electron transfer, enabling sensitive and rapid detection across broad ranges (1 nM–1,000 µM for superoxide and 1 nM to 850 µM for nitric oxide) with low detection limits (0.27 nM and 0.34 nM, respectively). The Ag-MOF sensor demonstrated high efficacy in tracking superoxide and nitric oxide from HepG2 and RAW 264.7 cells, as well as exogenous NO from chemical donors, making it suitable for dynamic biomarker monitoring in pathological studies. Tested in real biological fluids, it achieved high accuracy (94.10%–99.57% recovery), offering a reliable platform for applications in extracellular vesicle (EV) biosensors and disease diagnostics (Arul et al., 2024).

In their recent Joshi and Slaughter (2024) study, developed a highly sensitive electrochemical detection method for uric acid (UA) using multiwalled carbon nanotube (MWCNT)-supported iron nanostructured interfaces (FeNS/MWCNT). This one-step electroreduction approach allowed precise control over electrode synthesis by adjusting deposition potentials, bath solution pH, and growth times to optimize the electrochemical response. The Fe nanostructures, uniformly deposited on MWCNTs, demonstrated high sensitivity across a wide linear detection range (5–500 μM) and a low detection limit of 3.26 μM. The combination of FeNS and MWCNTs enhanced electrocatalytic activity by increasing active surface area, which facilitated sensitive, reliable biomarker detection. Although it applied to UA, this methodology provides valuable insights for electrochemical detection of (EVs) in biosensing. Like UA, EVs require precise, low-detection limit sensing due to their importance in cellular communication and disease diagnostics. MWCNT-supported nanostructures, such as those developed in this study, offer scalable platforms for EV detection by enabling stable, high-sensitivity electrochemical interfaces, which could be adapted to capture EV-specific biomarkers effectively in complex biological fluids. This research highlights potential avenues for designing nanostructured, electrocatalytic biosensors tailored to EV analysis, paving the way for non-invasive diagnostics and real-time health monitoring.

Recently Zhang et al. (2024) introduced an ultrasensitive electrochemical biosensor based on an ion-sensitive field-effect transistor (ISFET) for detecting cardiac troponin I (cTnI), a critical biomarker for acute myocardial infarction. This ISFET device, enhanced with Prussian blue-gold nanoparticles, achieves rapid detection within minutes, displaying an impressive sensitivity range (1–1,000 pg/mL) and an ultralow detection limit of 0.3 pg/mL. The sensor’s large sensing area and fast response make it ideal for emergency diagnostic applications where immediate biomarker quantification is essential. This platform exemplifies how electrochemical detection methods can be adapted for real-time and point-of-care diagnostics, especially relevant for extracellular vesicle (EV) applications, where rapid and sensitive detection of biomolecular changes in complex samples is crucial. By adjusting similar sensor designs for EV biomarkers, such technologies could enhance diagnostic precision in cancer and cardiovascular disease by capturing disease-specific signals released by EVs. Also, Lopez Baltazar et al. (2024) developed a surface plasmon resonance (SPR) biosensor for detecting extracellular vesicles (EVs) by targeting CD81, a transmembrane protein biomarker critical in EV-based diagnostics. By immobilizing polyclonal antibodies on mixed self-assembled monolayers (SAMs) of oligo ethylene glycol (OEG) with carboxylic and hydroxyl groups, the team optimized antibody surface coverage and clonality to enhance EV binding efficiency. They identified that a 40% coverage of polyclonal antibodies linked to a SAM with 10% carboxyl groups provided optimal sensitivity, achieving a detection limit of 5.9 × 1,065.9 \times 10^65.9 × 106 EVs/mL across a linear range of 1.9 × 1,081.9 \times 10^81.9 × 108–1.9 × 1,091.9 \times 10^91.9 × 109 EVs/mL. This configuration yielded a high affinity for EVs (1.92 nM equilibrium dissociation constant), allowing reliable quantification despite cellular variability. This SPR-based immunoassay highlights the role of surface chemistry in creating precise, reproducible EV detection methods, potentially advancing disease diagnostics. This configuration, with a high affinity for EVs (1.92 nM equilibrium dissociation constant), allows for accurate quantification despite cellular variability, highlighting the importance of surface chemistry in developing precise, reproducible SPR-based immunoassays that could advance disease diagnostics.

Lastly, Wu et al. (2024b) developed a novel label-free colorimetric biosensor for exosome detection was developed using sporopollenin microcapsules (SP) as a natural substrate to support gold nanoparticles (AuNPs). By modifying the SP-Au complex with CD63 aptamers, the team designed the sensor to detect exosomes via catalytic color change. When exosomes are absent, the SP-Au complex catalyzes the oxidation of tetramethylbenzidine (TMB), producing a visible blue color; however, the presence of exosomes inhibits this catalytic activity as they bind to the AuNPs, blocking the reaction. This inhibition-based detection allowed the sensor to achieve a low detection limit of 10 particles/µL with a wide linear range (10–108 particles/µL). Notably, the biosensor demonstrated high resistance to protein interference and maintained stability in challenging environments, supporting its potential for reliable exosome-based diagnostics in clinical settings.

## 6 Mass-based detection

Mass-based detection of EVs is a sophisticated approach that involves isolating and characterizing these vesicles by leveraging their mass and associated molecular profiles, primarily through advanced mass spectrometry (MS) techniques. This process typically starts with EV isolation from biological fluids such as plasma, urine, or saliva, using methods like ultracentrifugation, size exclusion chromatography, or immunoaffinity capture (Burton et al., 2023). Once isolated, EVs are subjected to proteomic analysis where MS identifies and quantifies the protein contents of the vesicles (Subedi et al., 2021).

**FIGURE 11:**
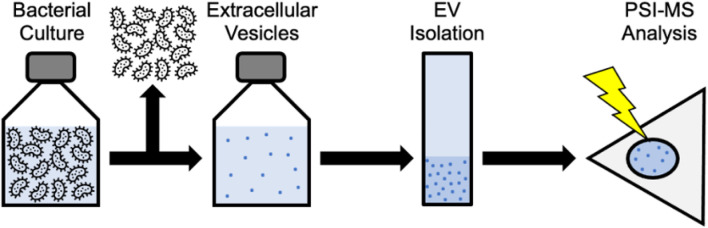
Represents the isolations and purification of Evs from bacterial culture supernatant tracked by PSI-MS. Reproduced from ref Chamberlain et al. (2021), with copyright permission under the terms of the CC-BY-NC-ND 4.0 license.

Furthermore, tandem mass spectrometry enables a detailed characterization of these EV proteins, offering valuable insights into their functional roles and their potential as biomarkers for various diseases, as illustrated in Figure 11. The high sensitivity and specificity inherent in MS allow researchers to detect minute quantities of EV proteins. This capability is particularly crucial for early disease diagnosis and for monitoring disease progression or treatment response (Bamford et al., 2023). For instance, titanium dioxide microspheres and gradient ultracentrifugation enhance the purity and yield of EVs, reducing co-isolation of contaminants and enabling accurate downstream MS analysis (Verkhoturov et al., 2021) Consequently, this mass-based approach not only provides a comprehensive overview of the EV proteome but also facilitates the discovery of novel biomarkers for a wide array of diseases, thereby advancing both diagnostic and therapeutic applications (Burton et al., 2023). Building upon this foundational understanding, several recent studies have highlighted significant advancements in the isolation and detection of EVs using mass-based methods. For example, Santiago et al. (2024) described an innovative method for isolating EVs using titanium dioxide (TiO2) microspheres. This technique leverages the selective interaction between TiO2 and EV membrane phospholipids, which effectively reduces the co-isolation of plasma proteins.

In their workflow, the EVs are initially precipitated from plasma using a precipitation agent, then further enriched by TiO_2_ microspheres, enhancing purity and yield. The enriched EVs were then subjected to mass spectrometry-based proteomics for detailed protein characterization, which allows for the identification of low-abundance EV proteins that are critical for diagnostic applications. This method offers a comprehensive workflow for the efficient isolation and characterization of plasma EVs, thereby facilitating their use in biomarker discovery and disease diagnostics.

Similarly, another example, Abyadeh et al. (2024) utilized tandem mass tag (TMT) quantitative proteomics to analyze mesenchymal stem cell (MSC)-derived EVs isolated through three different methods: ultracentrifugation (UC), high-speed centrifugation (HS), and ultracentrifugation on a sucrose cushion (SU). EVs were characterized for marker expression, size distribution, and morphology, followed by bioinformatic analysis of the proteome. The results revealed distinct molecular and functional characteristics based on the isolation method, with HS-EVs showing higher levels of ribosomal and mitochondrial proteins, while SU and UC-EVs had proteins involved in immune response and cell interactions. This study underscored the critical importance of selecting appropriate isolation methods tailored to specific research or clinical applications, as different techniques yield EVs with varying protein profiles and biological functions.

Further refining isolation techniques, Sharma and Dhamija (2024) who developed an improved ultracentrifugation method to isolate small extracellular vesicles (sEVs) from biofluids like plasma, saliva, and urine. They employed gradient ultracentrifugation and ultrafiltration to achieve high purity and yield of sEVs. After isolation, they conducted mass spectrometry-based shotgun proteomics and RNA isolation to profile the protein and RNA contents of the sEVs. This method enabled detailed characterization of the molecular cargo within sEVs, providing valuable insights for disease diagnosis and research.

Comparisons between different isolation strategies were also a focus for Askeland et al. (2020). , who used mass spectrometry to compare EVs isolated by high-speed centrifugation (HS), size exclusion chromatography (SEC), and peptide-affinity precipitation (PAP/ME kit). Their study evaluated the abundance, subtypes, and contamination levels of EVs using nanoparticle tracking analysis, immunoblotting, and transmission electron microscopy. Label-free tandem mass spectrometry was employed to analyze the proteome, revealing that HS and SEC methods resulted in higher EV abundance but also higher contamination, while PAP had lower EV abundance and higher contamination levels. In contrast, the PAP method produced a lower abundance of EVs but also exhibited a higher level of contamination. Consequently, the study determined that both HS and SEC are appropriate for MS biomarker studies, with the optimal choice contingent upon the concentration on larger or smaller EVs.

In the context of disease application, Li D. et al. (2024) introduced a novel EVID-biochip (EVs identification and detection biochip) that employed a mass-based detection method alongside immunomagnetic beads to isolate and quantify L1CAM-positive EVs, specifically those linked to Parkinson’s disease. This biochip system uses CD81 antibody-coated magnetic beads to capture neuronal EVs, relying on the specific binding of the neuronal marker L1CAM. The mass of these antibody-bound EVs is then measured through an integrated electrochemical protein detection system, which provides sensitive, mass-dependent quantification. This setup achieved a sensitivity of 1 pg/mL for L1CAM, successfully distinguishing Parkinson’s patients from controls with an area under the curve (AUC) of 0.973. This EVID-biochip allows efficient EV isolation and detection in a small sample volume (300 µL) within a 1.5-h assay time, demonstrating its potential for rapid and reliable biomarker discovery in neurological diseases, Expanding the diagnostic utility of EV analysis, Lozano et al. (2023) used mass-based detection methods to detect Trypanosoma cruzi infection from EVs in serum for diagnosing chronic Chagas disease. EVs in this context carry cell-free DNA (cfDNA) and exovesicle DNA specific to the Trypanosoma cruzi parasite, allowing for the detection of active infections even in patients with low parasitemia. The team specifically targeted the parasite’s mitochondrial kinetoplast DNA (kDNA) and nuclear satellite DNA (SAT) regions using nested and quantitative PCR (qPCR). Through this method, EVs from patient serum were identified as “containers” of T. cruzi DNA, which were subsequently amplified and quantified, achieving a high sensitivity. This mass-based detection confirmed active parasitic presence in asymptomatic carriers or those with modest parasitemia, opening a novel path for parasitic infection diagnosis using EVs and cfDNA indicators.

Finally, demonstrating the synergy of advanced analytical techniques and computational power, Bamford et al. (2023) utilized Time-of-Flight Secondary Ion Mass Spectrometry (ToF-SIMS) coupled with machine learning (ML) to identify and analyze extracellular vesicles (EVs) associated with neuroinflammation. This technique allowed for high-resolution, mass-based detection of chemical variations in EVs derived from microglia under stress conditions, requiring only 1 µL of sample. Integration of ToF-SIMS and ML integration enabled precise differentiation between EVs from LPS-stimulated microglia and controls, highlighting a significant reduction in free cysteine thiols in the treated samples, a marker of oxidative stress. This study showcases the potential of ToF-SIMS and ML as a highly sensitive tool for exploring disease-related molecular profiles in EVs.

## 7 Current state-of-the-art devices for point-of-care diagnostics

Beyond the selection of aptamers, they hold significant clinical translational relevance due to their compact structure, robustness, and compatibility with user-friendly biosensors. Several cutting-edge innovations have emerged in this field, including: Lab-on-a-Chip (LoC) Systems: Aptamer-based detection systems are increasingly integrated with microfluidic chips on a single platform, enabling the analysis of minimal sample volumes with rapid assay times (Surappa et al., 2023). Nanomaterial-Based Aptasensors: These aptasensors enable effective detection of metals by incorporating materials such as graphene oxide and gold nanoparticles, which enhance detection signals while improving stability, ease of production, and cost-effectiveness (Azzouz et al., 2023). Electrochemical Aptasensors: Aptamers play a crucial role in achieving high accuracy, sensitivity, and low detection limits with real-time monitoring capabilities. These sensors show great potential for portability, allowing integration into smartphone-based wearable devices that offer convenient operation and stability (Villalonga et al., 2022). Additionally, the integration of SELEX with AI-based analysis provides numerous advantages, including faster discovery of high-affinity extracellular vesicle (EV) aptamers for therapeutic monitoring and precision medicine applications.

## 8 Conclusions and future perspectives

Extracellular vesicles (EVs) have recently gained attention due to emerging research highlighting their therapeutic potential. Moreover, their convenient, non-invasive sampling and multiple ways of isolation and characterization. Therefore, coming up with methods for detection that are easy and cheap is mandatory to facilitate the research process. This review discusses the detection methods available such as the traditional EV detection, biosensors and antibody-based biosensors. These methods come with limitations; however, aptamers are now a promising alternative to detect exosomes due to their small size, high affinity, stability and easy chemical modifications. With the various methods available to use aptamers such as optical detection (SPR, FRET and fluorescence), nanoparticle-based detection, electrochemical detection and mass-based detection, further research and experiments are needed to integrate their use in practice and to overcome their limitations.

Although many primary EV quantification and characterization methods have developed and correlated with the diagnosis and prognosis of the disease, there are still significant gaps to be filled between basic EV research and clinical application. There are several challenges that have to be overcome. For example, the current EV isolation method, such as ultracentrifugation, cannot efficiently separate the specific EV subtypes. Aptamer-based EV isolation using EV membrane-specific targets would be more efficient. However, vesicle-free biomarkers coexisting in the sample is an obstacle to the specific recognition between the aptamer and the membrane protein the in the EVs. Although Aptamers-based systems are highly sensitive and selective, accurately quantifying and phenotyping vesicles is complex. Issues such as the “swarm effect” can lead to ambiguous results, where multiple vesicles may be mistaken as a single event. Another major limitation lies in the selection of aptamers specific to EVs, which is considerably more complex than selecting aptamers for purified proteins. Therefore, thorough validation and standardization protocols are essential to ensure the reliability and reproducibility of aptamers intended for EV targeting. Therefore, significant errors in the EV analysis occur. The future direction must be accurate methods that can target specific EVs. On the other hand, research should focus on the development of aptamers with improved binding affinity and specificity for exosome biomarkers, as well as integrating aptamers into portable, low-cost detection systems. Therefore, this could be achieved by integrating multiple available methods together.

The integration of nanomaterials, such as gold or silver nanoparticles or carbon nanotubes, with aptamer-based assays is another promising direction to enhance sensitivity and signaling. For example, aptasensor using palladium nanoparticle-modified metal-organic frameworks (MOFs) to improve sensitivity and minimize false positives when detecting exosomes. This approach demonstrated high sensitivity and selectivity for exosomes derived from different cell lines, with detection limits as low as 86.2 particles/µL (Li et al., 2023). A technique called APPROACH in which a combination of aptamer-mediated proximity ligation analysis (PLA) with rolling circle amplification (RCA) and time-resolved Förster resonance energy transfer (TR-FRET) was used for sensitive detection of exosomal biomarkers (Li Y. et al., 2024). Despite the enormous aptamer-based clinical research work that has been published, only a few aptamers have been successfully implemented for clinical application as of now. Aptamers are prone to rapid degradation by nucleases, resulting in short half-lives, highlighting the urgent need for strategies to enhance their stability. Furthermore, limited toxicological data on aptamers in humans raises safety concerns, particularly as few aptamer-based therapies have reached clinical application.

Next-generation aptamer-based biosensors hold significant therapeutic potential in the field of extracellular vesicles (EVs), offering promising solutions to overcome existing limitations in clinical translation. Future biosensors can be enhanced through the integration of nanomaterials such as graphene oxide, quantum dots, and gold nanoparticles, which improve both stability and sensitivity. Functionalized aptamers may further increase specificity for targeting disease-associated EVs, thereby enhancing their therapeutic relevance. Additionally, multiplexed aptasensors can improve diagnostic accuracy and specificity by effectively profiling heterogeneous EV populations. Moreover, the incorporation of artificial intelligence (AI) and machine learning into EV analysis represents a transformative advancement for data interpretation. The integration of bio sensing technologies with computational tools is expected to significantly enhance the clinical utility of aptamer-based applications.

Future directions should prioritize optimizing aptamers for targeting specific exosome subtypes and leveraging multiplexed assays for detailed biomarker profiling. An aptamer-based miniaturized instrument-free paper-based point-of-care colorimetric assay would be highly beneficial for patients residing in remote areas with limited laboratory access.

## Glossary

EVs: extracellular vesicles
sEVs: Small extracellular vesicles
tdEV: tumor-derived EV
DNA: deoxyribonucleic acid
RNA: Ribonucleic acid
SELEX: Systematic Evolution of Ligands by Exponential Enrichment
MVs: microvesicles
ARF6: Alternate reading frame −6
ERK: Extracellular signal-regulated kinase
MLCK: Myosin light-chain kinase
ROCK: Rho-associated protein kinases
ILVs: Intraluminal vesicles
Rab11: Ras-associated binding −11
MVBs: Multivesicular bodies
Tsp: Thrombospondin
ELISA: Enzyme-linked immunoassay
SPR: Surface plasmon resonance
QCM: Quartz crystal microbalance
HRP: Horseradish peroxidase
SPRi: Plasmon Resonance Imaging
HER2: Human epidermal growth factor receptor-2
PCR: Polymerase Chain Reaction
RT-PCR: Real-time- Polymerase Chain Reaction
MeORNA–HNA: 2′-O-methyl-ribose–1,5 anhydrohexitol nucleic acid
VEGF: Vascular Endothelial Growth Factor
**K**_**d**_: dissociation constant
FRET: Fluorescence resonance energy transfer
FP: Fluorescence polarization
FSA: Fluorescence signal amplification
EpCAM: Epithelial cellular adhesion molecule
GO: Graphene Oxide
CRC: colorectal cancer
HIV: Human immunodeficiency virus
BRCA: Branched rolling circle amplification
MUC1: Mucin 1
LOD: Limit of detection
hMFEX: Homogenous magneto-fluorescent exosome
LRET: Luminescence resonance energy transfer
UCNPs: Upconversion nanoparticles
TAMRA: Tetramethyl rhodamine
NMM: N-methylmesoporphyrin IX
ThT: thiosemicarbazone T
QDs: Quantum Dots
AuNps: Gold nanoparticles
SA-MB: Streptavidin-modified magnetic beads
AICHA: Aptamer-initiated catalytic hairpin assembly
FP/FA: Fluorescence Polarization/Fluorescence Anisotropy
hFCM: High-resolution flow cytometry
TMB: 3,30,5,50-tetramethylbenzidine
FITC: Fluorescein isothiocyanate
MAB: Molecular aptamer beacon
SERS: Surface-Enhanced Raman Scattering
AEX: anion exchange chromatography
HCR: Hybridization chain reaction
MOF: Metal organic framework
MWCNT: multiwalled carbon nanotube
SWCNT: Single walled carbon nanotube
ISFET: Ion-sensitive field-effect transistor
SP: Sporopollenin microcapsules
MS: Mass spectrometry
TMT: Tandem mass tag
MSC: Mesenchymal stem cell
SEC: Size exclusion chromatography
PAP: Peptide-affinity precipitation
AUC: Under the curve
ToF-SIMS: Time-of-Flight Secondary Ion Mass Spectrometry
PLA: Proximity ligation analysis
